# Neutrosophic Extension of the New Odd Weibull-Inverse Weibull Distribution: Theory and Applications

**DOI:** 10.12688/f1000research.172480.1

**Published:** 2025-12-26

**Authors:** Ahmed M. Salih, sara khalaf, Kamal N. Abdullah, Nooruldeen A. Noori

**Affiliations:** 1 mathematics, Tikrit University, Tikrit, Saladin Governorate, 34001, Iraq; 2 mathematics, University of Fallujah, Al-Fallujah, Al Anbar Governorate, 31002, Iraq

**Keywords:** Neutrosophic distributions, NNOWIW distribution, probability weighted moments, entropy measures, statistical estimation methods

## Abstract

This paper presents a Neutrosophic extension of the New odd Weibull inverse Weibull (NNOWIW) distribution, aiming to develop a statistical model capable of handling ambiguous or imprecise data. The mathematical formulation of the proposed distribution was derived by combining Neutrosophic logic with the T-X method, specifying the NCDF, the NPDF and the survival and hazard functions. The statistical probability of the distribution was analyzed. To achieve optimal estimation of the distribution parameters, the maximum likelihood (MLE), least square (LSE), and weighted least squares (WLSE) methods were used, and their performance was evaluated using Monte Carlo simulations. The models efficiency was also tested using real battery life data and compared to competing distributions. The results show that the proposed distribution shows competitive and flexible performance in terms of information criteria (AIC, CAIC, BIC, HQIC) and goodness-of-fit tests suggesting its potential usefulness for modeling complex data. However, further validation on larger and more diverse datasets is required to generalize these findings.

## 1. Introduction

Modeling failure and lifespan data is a fundamental topic in applied statistics, given its importance in various fields such as engineering, medicine, economics, and the environment. Among the classical distributions widely used in this field are the Weibull and inverse Weibull distributions, due to their flexibility in representing increasing and decreasing hazard functions. However, the limitations of these models in handling complex data have prompted many researchers to propose more sophisticated and flexible distributions.

One of the most prominent methods that contributed to the emergence of these new distributions is the T-X method, proposed by Alzaatreh et al. in 2013, ^1^ which opened the door to generating a large number of general-form distribution families based on basic distributions. This method has led to the development of a wide range of generative families, including: A modified T-X family, ^2^ A new logarithmic family, ^3^ Odd inverted Topp Leone-H family, ^4^ Shifted exponential-G family, ^5^ Generalized Odd Maxwell family, ^6^ Odd Lomax-G family, ^7^ hybrid Odd exponential- Φ. ^8^

Despite the wide variety of these generative distributional families, most of them assume that the data used are exact and precise. This means they do not account for ambiguity, indeterminacy, or partial information. This represents a clear knowledge gap in the field of modeling real-life data, especially in medical, industrial, environmental fields, where data are often incomplete, derived from expert estimates, or subject to ambiguity. Furthermore, these families, despite their flexibility, have not been used within a Neutrosophic logic framework, which weakens their ability to comprehensively handle uncertain or probable data.

The importance of this research stems from bridging this gap by combining the flexibility of statistical models generated by T-X method with the power of uncertainty representation provided by Neutrosophic logic, to construct an integrated probabilistic model capable of handling both ambiguous. The proposed distribution represents a step toward creating more realistic and accurate statistical tools for analyzing non-deterministic phenomena.

Unlike fuzzy logic, which primarily represents uncertainty by degrees of membership, and Bayesian approaches, which rely on prior assumptions, Neutrosophic logic explicitly incorporates truth (T), falsity (F), and indeterminacy (I) into the modeling framework. This three-component structure allows the proposed NNOWIW distribution to handle interval-valued parameters and contradictory information more effectively, which is particularly useful in real datasets with incomplete or imprecise observations. This distinctive feature provides an additional layer of flexibility and realism compared to traditional approaches, thereby strengthening the motivation for introducing the Neutrosophic extension.

This paper is organized as follows: Section 2 presents the mathematical formulation of the new Neutrosophic inverse Weibull distribution (NNOWIW), including the probability density function (NPDF), the cumulative distribution function (NCDF), and the survival and hazard functions. Section 3 covers the basic statistical properties of the proposed distribution, such as function expansions, the moment generating function MGF, and probability weighted moments PWM, as well as entropy measures (Renyi, Tsallis, Havrda-Charvat). Section 4 reviews parameter estimation method (MLE, LSE, and WLSE), presents the required derivatives of the maximum likelihood functions. Section 5 presents a Monte Carlo simulation study to evaluate the efficiency of the estimation methods according to metrics such as MSE, RMSE, and bias. Section 6 then highlights the practical applications of the proposed distribution by analyzing real battery life data and comparing it with competing distributions using statistical fit measures (AIC, BIC, HQIC) and goodness-of-fit tests. Finally, Section 7 concludes the paper with a summary of the main results and conclusions, indicating some potential avenues for future work.

## 2. The mathematical formulation of the NNOWIW distribution

The section discusses the mathematical representation of the NNOWIW distribution by presenting its basic components, including the CDF, the hazard function and survival function. Graphs illustrating the behavior of the understanding of the formal properties of the distribution. The CDF of the NNOWIW distribution is represented as follows:

Definition 1: Let the Neutrosophic random variable be represented as $X_{N}=d+t_{I}$ , where $t_{I}\in \left[X_{L},X_{U}\right]$ . The values $X_{L}$ and $X_{U}$ denoted the lower and upper bounds of the uncertain part of the random variable belonging to the new Neutrosophic odd Weibull family NOWG. This variable consists of a deterministic component d and an indeterminate $t_{I}\in \left[I_{L},I_{U}\right]$ .When the indeterminacy vanishes $X_{L}=X_{U}$ , the Neutrosophic model reduces to the classical new odd Weibull family. The NPDF and NCDF are characterized by the Neutrosophic shape parameter $\eta_{N}\in \left[\eta_{L},\eta_{U}\right]$ and $\zeta_{N}\in \left[\zeta_{L},\zeta_{U}\right]$ , and they take the following general form:

$$F_{\text{NNOWG}}\left(x_{N},\eta_{N},\zeta_{N},\varepsilon \right)=1-e^{\left(-\eta_{N}{\left[-H\left(x_{N},\varepsilon \right).\log\left(1-H\left(x_{N},\varepsilon \right)\right)\right]}^{\zeta_{N}}\right)}$$ (1)

$$f_{\text{NNOWG}}\left(x_{N},\eta_{N},\zeta_{N},\varepsilon \right)=\eta_{N}\zeta_{N}h\left(x_{N},\varepsilon \right)\left[\frac{H\left(x_{N},\varepsilon \right)}{1-H\left(x_{N},\varepsilon \right)}-\log\left(1-H\left(x_{N},\varepsilon \right)\right)\right]\times {\left[-H\left(x_{N},\varepsilon \right).\log\left(1-H\left(x_{N},\varepsilon \right)\right)\right]}^{\zeta_{N}-1}e^{\left(-\eta_{N}{\left[-H\left(x_{N},\varepsilon \right).\log\left(1-H\left(x_{N},\varepsilon \right)\right)\right]}^{\zeta_{N}}\right)}$$ (2)

Where $H\left(x_{N},\varepsilon \right)$ and $h\left(x_{N},\varepsilon \right)$ are NCDF and NPDF of baseline distribution with ε parameter. ^9^

Definition 2: Let the Neutrosophic random variable be represented as $X_{N}=d+t_{I}$ , where $t_{I}\in \left[X_{L},X_{U}\right]$ . The values $X_{L}$ and $X_{U}$ denote the lower and upper bounds of the uncertain part of the random variable belonging to the Neutrosophic inverse Weibull distribution (NIW). This variable consists of a deterministic component d and an indeterminate $t_{I}\in \left[I_{L},I_{U}\right]$ .When the indeterminacy vanishes $X_{L}=X_{U}$ , the Neutrosophic model reduces to the classical inverse Weibull distribution. The NPDF and NCDF are characterized by the Neutrosophic shape parameter $k_{N}\in \left[k_{L},k_{U}\right]$ and $n_{N}\in \left[n_{L},n_{U}\right]$ , and they take the following general form:

$$H\left(x_{N},k_{N},n_{N}\right)=e^{-k_{N}x_{N}^{-n_{N}}}$$ (3)

$$h\left(x_{N},k_{N},n_{N}\right)=k_{N}n_{N}x_{N}^{-\left(n+1\right)}e^{-k_{N}x_{N}^{-n_{N}}}$$ (4)

Where $x_{N},k_{N},n_{N}>0$ such as $x_{N}$ is Neutrosophic random variable, and $k_{N},n_{N}$ are neutrosophic shape parameters.

The NCDF of the proposed NNOWIW distribution is derived through appropriate substitution into the general form Equation (3) into (1) as follows:

$$F_{\text{NNOWIW}}\left(x_{N},\eta_{N},\zeta_{N},k_{N},n_{N}\right)=1-e^{\left(-\eta_{N}{\left[-e^{-k_{N}x_{N}^{-n_{N}}}.\log\left(1-e^{-k_{N}x_{N}^{-n_{N}}}\right)\right]}^{\zeta_{N}}\right)}$$ (5)

The NPDF of the NNOWIW distribution is derived either by differentiating Equation (5) or by substituting Equations (4) and (3) into Equation (2), as detailed below

$$f_{\text{NNOWG}}\left(x_{N},\eta_{N},\zeta_{N},k_{N},n_{N}\right)=\eta_{N}\zeta_{N}k_{N}n_{N}x_{N}^{-\left(n+1\right)}e^{-k_{N}x_{N}^{-n_{N}}}\left[\frac{e^{-k_{N}x_{N}^{-n_{N}}}}{1-e^{-k_{N}x_{N}^{-n_{N}}}}-\log\left(1-e^{-k_{N}x_{N}^{-n_{N}}}\right)\right]\times {\left[-e^{-k_{N}x_{N}^{-n_{N}}}.\log\left(1-e^{-k_{N}x_{N}^{-n_{N}}}\right)\right]}^{\zeta_{N}-1}e^{\left(-\eta_{N}{\left[-e^{-k_{N}x_{N}^{-n_{N}}}.\log\left(1-e^{-k_{N}x_{N}^{-n_{N}}}\right)\right]}^{\zeta_{N}}\right)}$$ (6)

The following expression is used to derive the Neutrosophic survival function, as stated in Ref. 10:

$$S\left(x\right)=e^{\left(-\eta_{N}{\left[-e^{-k_{N}x_{N}^{-n_{N}}}.\log\left(1-e^{-k_{N}x_{N}^{-n_{N}}}\right)\right]}^{\zeta_{N}}\right)}$$ (7)

The calculation of the neutrosophic hazard functions for the NNOWIW distribution is based on the following formula, as presented in the Ref. 11:

$$h\left(x\right)=\eta_{N}\zeta_{N}k_{N}n_{N}x_{N}^{-\left(n+1\right)}e^{-k_{N}x_{N}^{-n_{N}}}\left[\frac{e^{-k_{N}x_{N}^{-n_{N}}}}{1-e^{-k_{N}x_{N}^{-n_{N}}}}-\log\left(1-e^{-k_{N}x_{N}^{-n_{N}}}\right)\right]\times {\left[-e^{-k_{N}x_{N}^{-n_{N}}}.\log\left(1-e^{-k_{N}x_{N}^{-n_{N}}}\right)\right]}^{\zeta_{N}-1}$$ (8)

**Figure 1.**
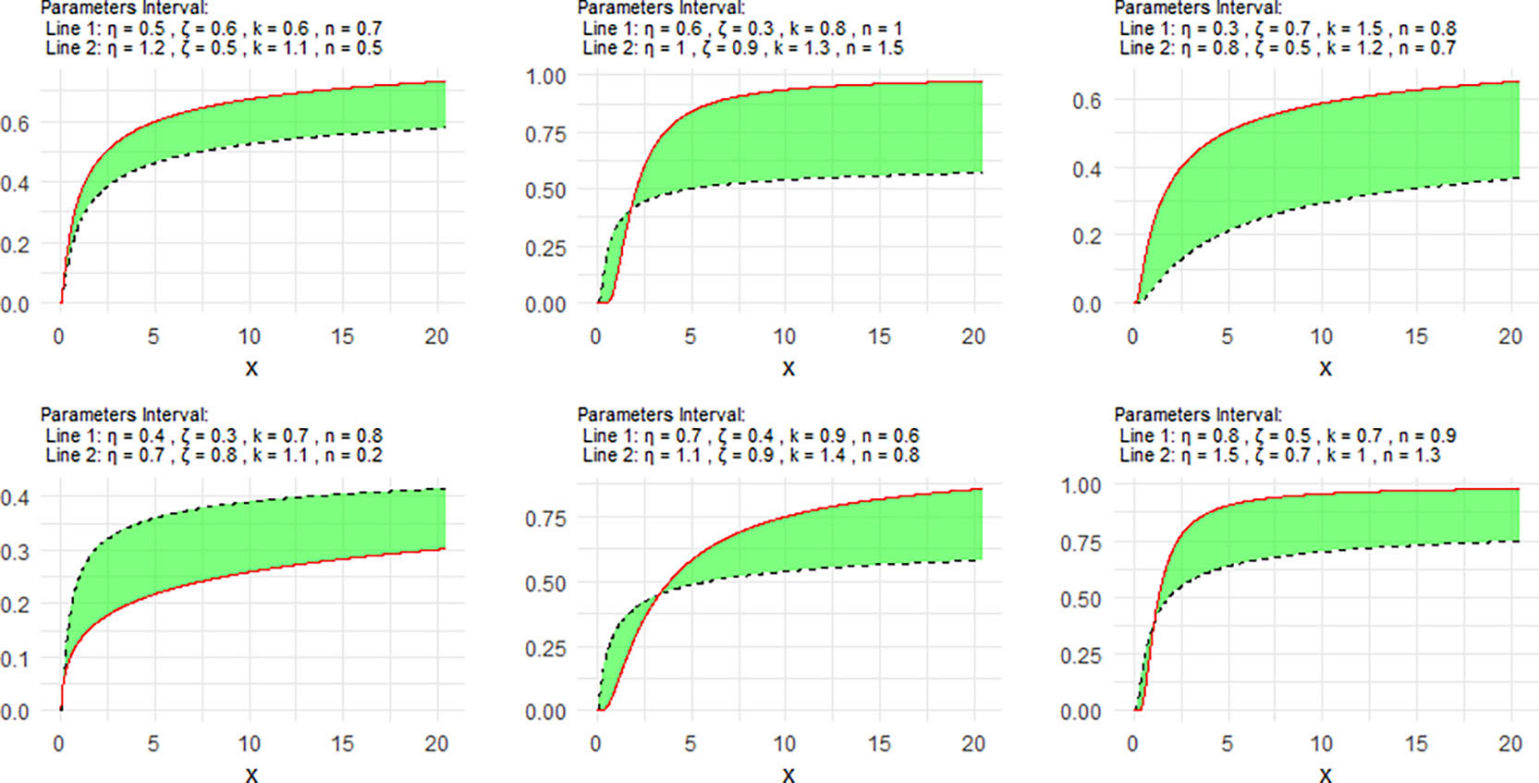
NCDF for NNOWIW distribution.

**Figure 2.**
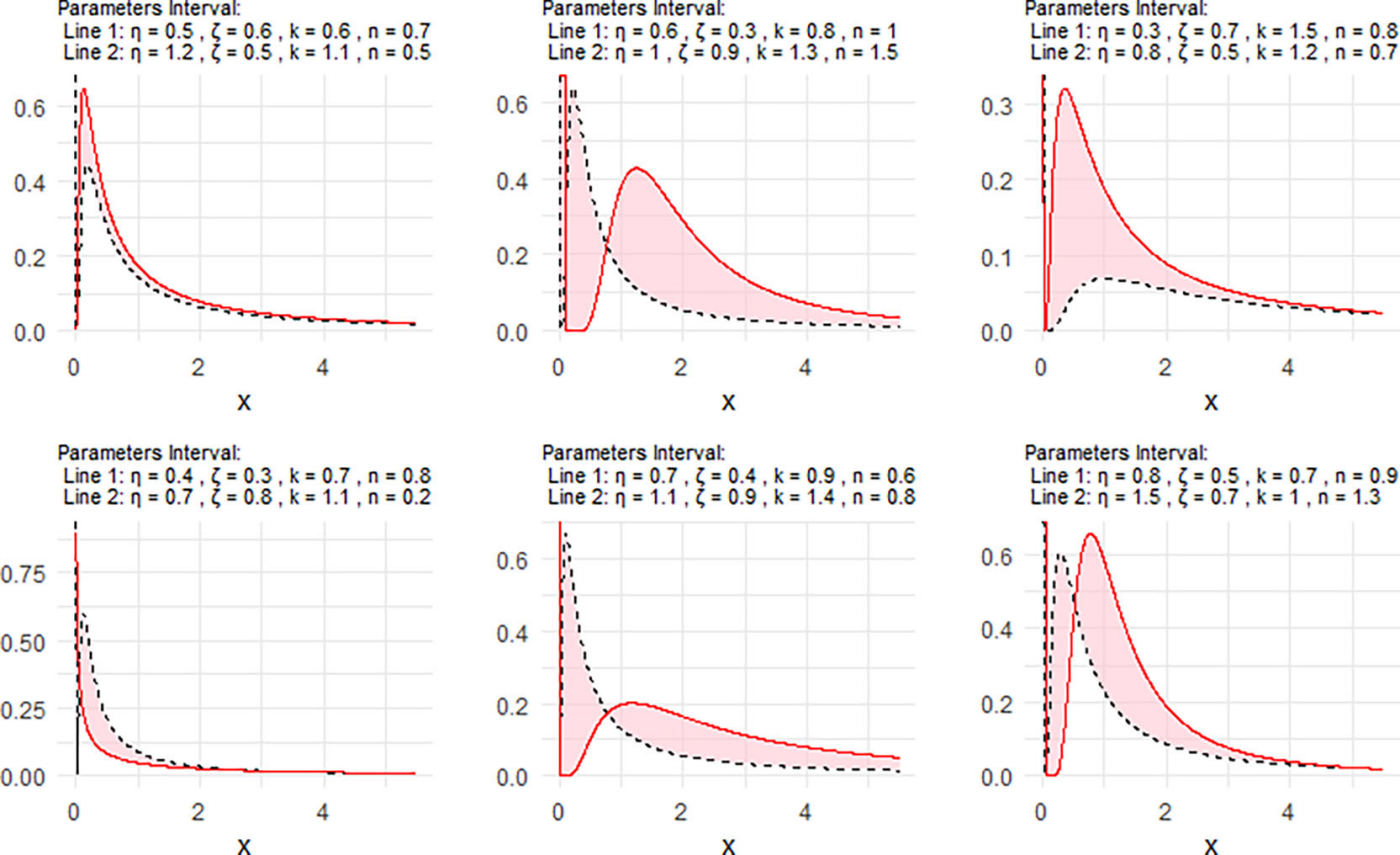
NPDF for NNOWIW distribution.

**Figure 3.**
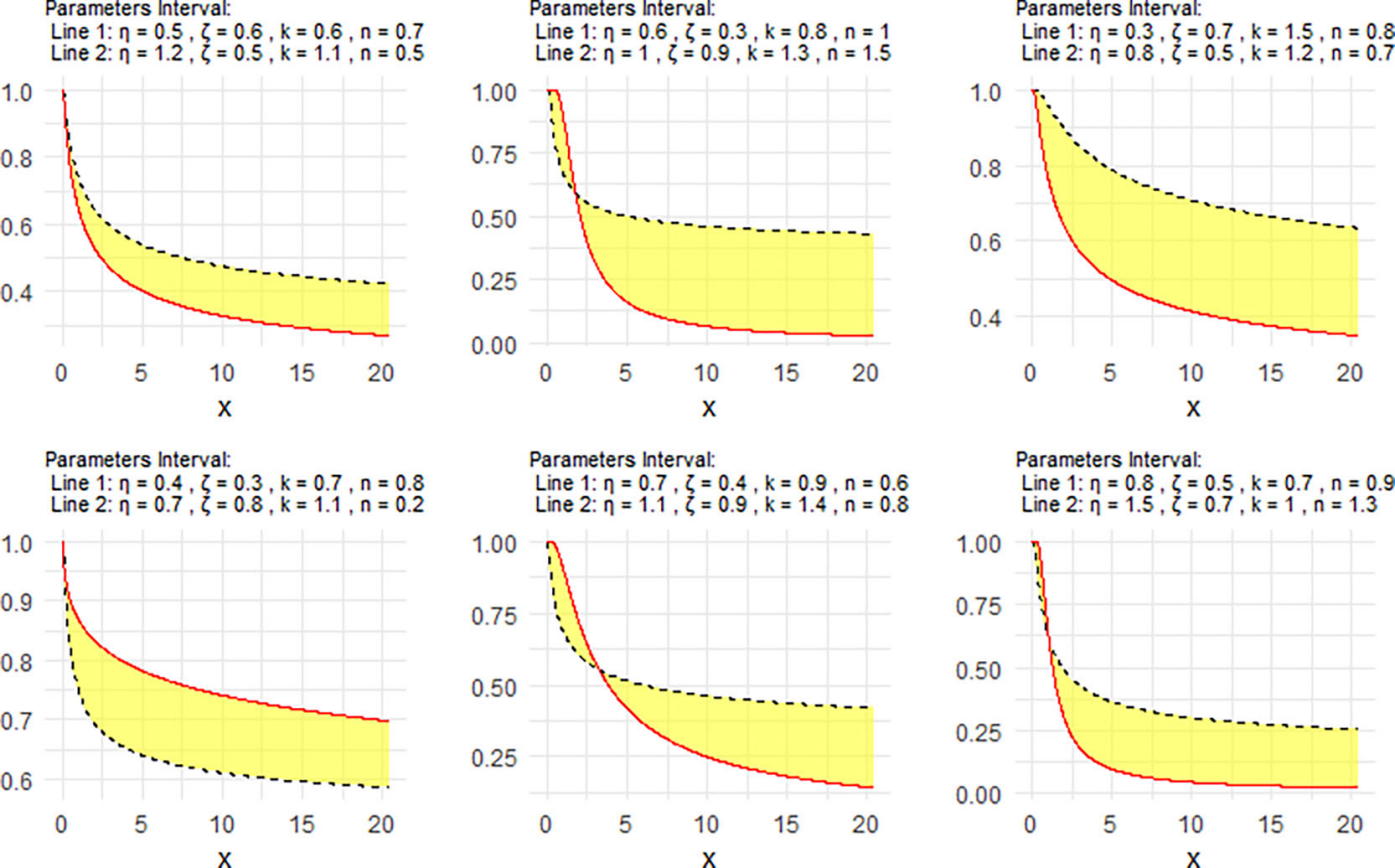
Survival of NNOWIW distribution.

Figure 1 includes a representation of the NCDF of the NNOWIW distribution, using variable intervals for its Neutrosophic coefficients. Figure 2 includes a representation of the NPDF of the NNOWIW distribution, using variable intervals for its Neutrosophic coefficients. Figure 3 shows the Neutrosophic survival function (NSF) for the NNOWIW distribution, plotted using different values for its Neutrosophic coefficients.

## 3. Statistical properties

This section provides a detailed examination of the statistical properties of the NNOWIW distribution, beginning with the function expansions of both the NCDF and NPDF, followed by the derivation of the quantile function. These expansions facilitate the extraction of several key statistical measures, including the non-central moments, moment generated function, incomplete moment, Lorenzo and Bonferroni curves, probability weighted moments, the characteristic function, and entropy measures.

### 3.1 NCDF and NPDF expansion

Due to the mathematical complexity of the NCDF and NPDF in Equations (5) and (6), respectively, both functions have been simplified to facilitate the analysis and derivation of the statistical properties of the NNOWIW distribution. This simplification is based on the binomial expansion, exponential function expansion, and logarithmic expansion. Accordingly, the simplified form of the NCDF is obtained as follows:

$$\begin{array}{c} F\left(x_{N}\right)=1-\vartheta e^{-\left(j_{N}+2i_{N}\zeta_{N}\right)k_{N}x_{N}^{-n_{N}}}\\ \text{Where}\;\vartheta =\sum_{i_{N}=j_{N}=0}^{\infty }\frac{{\left(-1\right)}^{i_{N}+i_{N}\zeta_{N}+j_{N}}}{i_{N}!}\eta_{N}^{i_{N}}d_{i_{N}\zeta_{N},j_{N}},\;\text{and}\;d_{i_{N}\zeta_{N},j_{N}}=j_{N}^{-1}\sum_{m=1}^{j_{N}}\frac{m\left(i_{N}\zeta_{N}+1\right)-j_{N}}{m+1}\;\text{for}\;j_{N}\geq 0\;\text{and}\;d_{i_{N}\zeta_{N},0}=1 \end{array}$$ (9)

Similarly, the NPDF can be expanded to derive the following form:

$$f\left(x_{N}\right)=ϒx_{N}^{-\left(n_{N}+1\right)}e^{-\left(2i_{N}\zeta_{N}+2\zeta_{N}+j_{N}+p_{N}\right)k_{N}x_{N}^{-n_{N}}}-\Phi x_{N}^{-\left(n_{N}+1\right)}e^{-\left(2i_{N}\zeta_{N}+2\zeta_{N}+j_{N}+z_{N}\right)k_{N}x_{N}^{-n_{N}}}$$ (10)

Where

$$ϒ=\sum_{i_{N}=j_{N}=p_{N}=0}^{\infty }\frac{{\left(-1\right)}^{i_{N}+i_{N}\zeta_{N}+\zeta_{N}-1+j_{N}+p_{N}}}{i_{N}!}\eta_{N}^{i_{N}+1}d_{i_{N}\zeta_{N}+\zeta_{N}-1,j_{N}}\zeta_{N}k_{N}n_{N}$$

And

$$\Phi =\sum_{i_{N}=j_{N}=Z_{N}=0}^{\infty }\frac{{\left(-1\right)}^{i_{N}+i_{N}\zeta_{N}+\zeta_{N}-1+j_{N}+Z_{N}}}{i_{N}!}\eta_{N}^{i_{N}+1}d_{i_{N}\zeta_{N}+\zeta_{N}-1,j_{N}}\zeta_{N}k_{N}n_{N}$$

As

$$d_{1,z_{N}}=z_{N}^{-1}\sum_{m=1}^{j_{N}}\frac{2m-j_{N}\;}{m+1}\;\text{for}\;z_{N}\geq 0\;\text{and}\;d_{1,0}=1$$

And

$$d_{i_{N}\zeta_{N},j_{N}}=j_{N}^{-1}\sum_{m=1}^{j_{N}}\frac{m\left(i_{N}\zeta_{N}+1\right)-j_{N}}{m+1}\;\text{for}\;j_{N}\geq 0\;\text{and}\;d_{i_{N}\zeta_{N},0}=1$$

### 3.2 The quantile function

The quantile function is one of the fundamental tools in analyzing the statistical properties of the NNOWIW distribution. It’s obtained by inverting the NCDF, which enables Monte Carlo simulation and provides deeper insights into distributional characteristics such as skewness, kurtosis, and the median. For the NNOWIW distribution, the quantile function Q(F) is derived by solving the equation $F\left(Q\left(F\right)\right)=b_{N}$ where $b_{N}$ is a probability value within the open interval (0,1). This function is expressed as $Q\left(b_{N}\right)=F^{-1}\left(b_{N}\right)$ , and it is derived for the NNOWIW distribution as follows ^12, 13^:

$$\mathrm{QF}={\left[\frac{\log\left[\frac{{\left[\frac{-\log\left(1-b_{N}\right)}{\eta_{N}}\right]}^{\frac{1}{\zeta_{N}}}}{{\left[\frac{-\log\left(1-b_{N}\right)}{\eta_{N}}\right]}^{\frac{1}{\zeta_{N}}}+W_{-1}{\left[\frac{-\log\left(1-b_{N}\right)}{\eta_{N}}\right]}^{\frac{1}{\zeta_{N}}}e^{{\left[\frac{-\log\left(1-b_{N}\right)}{\eta_{N}}\right]}^{\frac{1}{\zeta_{N}}}}}\right]}{k_{N}}\right]}^{\frac{-1}{n_{N}}}$$ (11)

Where *W*(.) refers to the Lombard function.

Table 1 shows the values of the quantile function at different intervals parameters.

**Table 1.** Quantile values for selected parameters of the NNOWIW distribution.

$s_{N}$	( $\eta_{N},\zeta_{N},k_{N},n_{N}$ )				
	[0.3,1,3], [1.7,2.7], [0.5,1.5], [0.8,1.8]	[0.4,1.4], [1.5,2.5], [0.4,1.4], [0.7,1.7]	[0.5,1.5], [1.6,2.6], [0.8,1.8], [0.6,1.6]	[0.2,1.2], [1.4,2.4], [0.7,1.7], [0.9,1.9]	[0.8,2.8], [1.9,2.9], [0.9,1.9], [0.5,1.5]
0.1	[0.95815, 1.60399]	[0.52946, 1.52535]	[1.38501, 1.84166]	[1.60076, 1.62682]	[1.67147, 2.02330]
0.2	[1.51982, 1.77943]	[0.89207, 1.70852]	[2.06871, 2.37971]	[1.81078, 2.8197]	[2.26463, 2.78811]
0.3	[1.91413, 2.17968]	[1.34554, 1.85107]	[2.24499, 3.60887]	[1.9542, 4.54820]	[2.44921, 4.06494]
0.4	[2.03432, 3.02764]	[1.96309, 1.97972]	[2.40373, 5.24985]	[2.08383, 7.25026]	[2.61333, 5.63795]
0.5	[2.15078, 4.19339]	[2.10565, 2.86448]	[2.55877, 7.58194]	[2.21102, 11.86537]	[2.77175, 7.68571]
0.6	[2.27117, 5.92107]	[2.23713, 4.29272]	[2.72025, 11.15010]	[2.34409, 20.66243]	[2.9348, 10.51989]
0.7	[2.40428, 8.76149]	[2.38404, 6.83650]	[2.90014, 17.21563]	[2.49320, 40.31431]	[2.93482, 14.78851]
0.8	[2.5657, 14.30078]	[2.56443, 12.34952]	[3.12033, 29.52009]	[2.67694, 97.64122]	[3.33080, 22.17841]
0.9	[2.80027, 29.88561]	[2.83047, 30.68424]	[3.44352, 66.19065]	[2.9492, 398.04552]	[3.64266, 39.41633]

### 3.3 Non-center moments

The non-central moments are fundamental statistics that provide deep insight into the distribution’s key characteristics, including measures of central tendency, variability, skewness, and kurtosis. These moments are essential for both theoretical developments and practical applications, as they help quantify the shape and behavior of the proposed NNOWIW distribution. The $r^{\mathrm{th}}$ non-central moment is defined as the expected value of $x^{r}$ , and for the NNOWIW model, it is derived as follows ^14– 16^:

$$\begin{array}{l} \mu_{r}=E{\left(x_{N}^{r}\right)}_{\text{NNOWIW}}=\int_{-\infty }^{\infty }x_{N}^{r}f\left(x_{N}\right)dx_{N}\\ \mu_{r}=ϒ\int_{0}^{\infty }x_{N}^{r-\left(n_{N}+1\right)}e^{-\left(2i_{N}\zeta_{N}+2\zeta_{N}+j_{N}+p_{N}\right)k_{N}x_{N}^{-n_{N}}}dx_{N}\\ \;-\Phi \int_{0}^{\infty }x_{N}^{r_{N}-\left(n_{N}+1\right)}e^{-\left(2i_{N}\zeta_{N}+2\zeta_{N}+j_{N}+z_{N}\right)k_{N}x_{N}^{-n_{N}}}dx_{N}\\ \mu_{r}=ϒI_{1}-\Phi I_{2} \end{array}$$

where

$$\begin{array}{c} I_{1}=\int_{0}^{\infty }x_{N}^{r-\left(n_{N}+1\right)}e^{-\left(2i_{N}\zeta_{N}+2\zeta_{N}+j_{N}+p_{N}\right)k_{N}x_{N}^{-n_{N}}}dx_{N}\\ I_{2}=\int_{0}^{\infty }x_{N}^{r-\left(n_{N}+1\right)}e^{-\left(2i_{N}\zeta_{N}+2\zeta_{N}+j_{N}+z_{N}\right)k_{N}x_{N}^{-n_{N}}}dx_{N} \end{array}$$

For $I_{1}$ , let

$$\begin{array}{l} \;y=\left(2i_{N}\zeta_{N}+2\zeta_{N}+j_{N}+p_{N}\right)k_{N}x_{N}^{-n_{N}}\\ I_{2}=\int_{0}^{\infty }x_{N}^{r-\left(n_{N}+1\right)}e^{-\left(2i_{N}\zeta_{N}+2\zeta_{N}+j_{N}+z_{N}\right)k_{N}x_{N}^{-n_{N}}}dx_{N}\\ \;x=\frac{y^{\frac{1}{n_{N}}}}{k_{N}^{\frac{1}{n_{N}}}{\left(2i_{N}\zeta_{N}+2\zeta_{N}+j_{N}+p_{N}\right)}^{\frac{1}{n}}}\;⟹\;dx_{N}=\frac{y^{\frac{1}{n_{N}}-1}}{n_{N}k_{N}^{\frac{1}{n_{N}}}{\left(2i_{N}\zeta_{N}+2\zeta_{N}+j_{N}+p_{N}\right)}^{\frac{1}{n}}}\mathrm{dy} \end{array}$$

Then

$$I_{1}=\int_{0}^{\infty }{\left(\frac{y^{\frac{1}{n_{N}}}}{k_{N}^{\frac{1}{n_{N}}}{\left(2i_{N}\zeta_{N}+2\zeta_{N}+j_{N}+p_{N}\right)}^{\frac{1}{n}}}\right)}^{r-\left(n_{N}+1\right)}e^{-y}\frac{y^{\frac{1}{n_{N}}-1}}{n_{N}k_{N}^{\frac{1}{n_{N}}}{\left(2i_{N}\zeta_{N}+2\zeta_{N}+j_{N}+p_{N}\right)}^{\frac{1}{n}}}\mathrm{dy}\;$$

By simplify $I_{1}$ to get a final form as follows:

$$\begin{array}{l} I_{1}=\frac{1}{n_{N}k_{N}^{\frac{r-n_{N}}{n_{N}}}{\left(2i_{N}\zeta_{N}+2\zeta_{N}+j_{N}+p_{N}\right)}^{\frac{r-n_{N}}{n_{N}}}}\int_{0}^{\infty }y^{\frac{r-n_{N}}{n_{N}}-1}e^{-y}\mathrm{dy}\\ I_{1}=\frac{\Gamma \left(\frac{r-n_{N}}{n_{N}}\right)}{n_{N}k_{N}^{\frac{r-n_{N}}{n_{N}}}{\left(2i_{N}\zeta_{N}+2\zeta_{N}+j_{N}+p_{N}\right)}^{\frac{r-n_{N}}{n_{N}}}} \end{array}$$

By same way for $I_{2}$ to get a final form:

$$I_{2}=\frac{\Gamma \left(\frac{r-n_{N}}{n_{N}}\right)}{n_{N}k_{N}^{\frac{r-n_{N}}{n_{N}}}{\left(2i_{N}\zeta_{N}+2\zeta_{N}+j_{N}+z_{N}\right)}^{\frac{r-n_{N}}{n_{N}}}}$$

To get:

$$\mu_{r}=\frac{\Gamma \left(\frac{r-n_{N}}{n_{N}}\right)}{n_{N}k_{N}^{\frac{r-n_{N}}{n_{N}}}}\left[\frac{ϒ}{{\left(2i_{N}\zeta_{N}+2\zeta_{N}+j_{N}+p_{N}\right)}^{\frac{r-n_{N}}{n_{N}}}}-\frac{\Phi }{{\left(2i_{N}\zeta_{N}+2\zeta_{N}+j_{N}+z_{N}\right)}^{\frac{r-n_{N}}{n_{N}}}}\right]$$ (12)

The first four moments are found by substituting the value of r and as follows

$$\mu_{1}=\frac{\Gamma \left(\frac{1-n_{N}}{n_{N}}\right)}{n_{N}k_{N}^{\frac{1-n_{N}}{n_{N}}}}\left[\frac{ϒ}{{\left(2i_{N}\zeta_{N}+2\zeta_{N}+j_{N}+p_{N}\right)}^{\frac{1-n_{N}}{n_{N}}}}-\frac{\Phi }{{\left(2i_{N}\zeta_{N}+2\zeta_{N}+j_{N}+z_{N}\right)}^{\frac{1-n_{N}}{n_{N}}}}\right]$$ (13)

$$\mu_{2}=\frac{\Gamma \left(\frac{2-n_{N}}{n_{N}}\right)}{n_{N}k_{N}^{\frac{2-n_{N}}{n_{N}}}}\left[\frac{ϒ}{{\left(2i_{N}\zeta_{N}+2\zeta_{N}+j_{N}+p_{N}\right)}^{\frac{2-n_{N}}{n_{N}}}}-\frac{\Phi }{{\left(2i_{N}\zeta_{N}+2\zeta_{N}+j_{N}+z_{N}\right)}^{\frac{2-n_{N}}{n_{N}}}}\right]$$ (14)

$$\mu_{3}=\frac{\Gamma \left(\frac{3-n_{N}}{n_{N}}\right)}{n_{N}k_{N}^{\frac{3-n_{N}}{n_{N}}}}\left[\frac{ϒ}{{\left(2i_{N}\zeta_{N}+2\zeta_{N}+j_{N}+p_{N}\right)}^{\frac{3-n_{N}}{n_{N}}}}-\frac{\Phi }{{\left(2i_{N}\zeta_{N}+2\zeta_{N}+j_{N}+z_{N}\right)}^{\frac{3-n_{N}}{n_{N}}}}\right]$$ (15)

$$\mu_{4}=\frac{\Gamma \left(\frac{4-n_{N}}{n_{N}}\right)}{n_{N}k_{N}^{\frac{4-n_{N}}{n_{N}}}}\left[\frac{ϒ}{{\left(2i_{N}\zeta_{N}+2\zeta_{N}+j_{N}+p_{N}\right)}^{\frac{4-n_{N}}{n_{N}}}}-\frac{\Phi }{{\left(2i_{N}\zeta_{N}+2\zeta_{N}+j_{N}+z_{N}\right)}^{\frac{4-n_{N}}{n_{N}}}}\right]$$ (16)

The skewness and kurtosis of the NNOWIW distribution are calculated respectively as follows ^17^:

$${\mathrm{SK}}_{\text{NNOWIW}}=\frac{\frac{\Gamma \left(\frac{3-n_{N}}{n_{N}}\right)}{n_{N}k_{N}^{\frac{3-n_{N}}{n_{N}}}}\left[\frac{ϒ}{{\left(2i_{N}\zeta_{N}+2\zeta_{N}+j_{N}+p_{N}\right)}^{\frac{3-n_{N}}{n_{N}}}}-\frac{\Phi }{{\left(2i_{N}\zeta_{N}+2\zeta_{N}+j_{N}+z_{N}\right)}^{\frac{3-n_{N}}{n_{N}}}}\right]}{{\left(\frac{\Gamma \left(\frac{2-n_{N}}{n_{N}}\right)}{n_{N}k_{N}^{\frac{2-n_{N}}{n_{N}}}}\left[\frac{ϒ}{{\left(2i_{N}\zeta_{N}+2\zeta_{N}+j_{N}+p_{N}\right)}^{\frac{2-n_{N}}{n_{N}}}}-\frac{\Phi }{{\left(2i_{N}\zeta_{N}+2\zeta_{N}+j_{N}+z_{N}\right)}^{\frac{2-n_{N}}{n_{N}}}}\right]\right)}^{\frac{3}{2}}}$$ (17)

$${\mathrm{KU}}_{\text{NNOWIW}}=\frac{\frac{\Gamma \left(\frac{3-n_{N}}{n_{N}}\right)}{n_{N}k_{N}^{\frac{3-n_{N}}{n_{N}}}}\left[\frac{ϒ}{{\left(2i_{N}\zeta_{N}+2\zeta_{N}+j_{N}+p_{N}\right)}^{\frac{3-n_{N}}{n_{N}}}}-\frac{\Phi }{{\left(2i_{N}\zeta_{N}+2\zeta_{N}+j_{N}+z_{N}\right)}^{\frac{3-n_{N}}{n_{N}}}}\right]}{{\left(\frac{\Gamma \left(\frac{2-n_{N}}{n_{N}}\right)}{n_{N}k_{N}^{\frac{2-n_{N}}{n_{N}}}}\left[\frac{ϒ}{{\left(2i_{N}\zeta_{N}+2\zeta_{N}+j_{N}+p_{N}\right)}^{\frac{2-n_{N}}{n_{N}}}}-\frac{\Phi }{{\left(2i_{N}\zeta_{N}+2\zeta_{N}+j_{N}+z_{N}\right)}^{\frac{2-n_{N}}{n_{N}}}}\right]\right)}^{2}}-3$$ (18)

Table 2 shows the values of first 4 moments, variance, skewness, and kurtoses at different intervals parameters.

**Table 2.** Intervals of selected moments of the NNOWIW distribution.

$\eta_{N}$	$\zeta_{N}$	$k_{N}$	$n_{N}$	${{\overset{`}{\mu }}_{1}}_{N}$	${{\overset{`}{\mu }}_{2}}_{N}$	${{\overset{`}{\mu }}_{3}}_{N}$	${{\overset{`}{\mu }}_{4}}_{N}$	$\sigma_{N}^{2}$	$S_{N}$	$K_{N}$
[0.6, 1.6]	[1.4, 2.4]	[0.5, 1.5]	[0.1,1.1]	[0.00145, 0.03546]	[0.00132, 0.01849]	[0.00121, 0.01248]	[0.00112, 0.00942]	[0.00132, 0.01723]	[4.96541, 25.2383]	[27.55528, 640.9644]
			[0.2,1.2]	[0.00146, 0.06442]	[0.00134, 0.03546]	[0.00123, 0.02432]	[0.00114, 0.01849]	[0.00134, 0.03130]	[3.64311, 25.19755]	[14.70541, 638.3678]
		[0.6, 1.6]	[0.3,1.3]	[0.00089, 0.06975]	[0.00082, 0.04188]	[0.00076, 0.02967]	[0.00071, 0.02291]	[0.00082, 0.03701]	[3.46154, 32.46292]	[13.06408, 1058.45]
			[0.4,1.4]	[0.00089, 0.08294]	[0.00083, 0.05250]	[0.00077, 0.03799]	[0.00073, 0.02967]	[0.00083, 0.04562]	[3.1588, 32.4317]	[10.76472, 1055.875]
	[1.8, 2.8]	[0.7, 1.7]	[0.5,1.5]	[0.00015, 0.05242]	[0.00014, 0.03757]	[0.00013, 0.02905]	[0.00012, 0.02360]	[0.00014, 0.03483]	[3.98889, 80.67237]	[16.71791, 6524.261]
			[0.6,1.6]	[0.00015, 0.05599]	[0.00014, 0.04156]	[0.00013, 0.03279]	[0.00013, 0.02699]	[0.00014, 0.03843]	[3.87069, 80.63411]	[15.62387, 6516.331]
		[0.9, 1.9	[0.7,1.7]	[4.57e-05, 0.027091]	[4.36e-05, 0.02143]	[4.17e-05, 0.01764]	[4e-05, 0.01494]	[4.36e-05, 0.02070]	[5.62074, 144.8847]	[32.52101, 21027.26]
			[0.8,1.8]	[4.58e-05, 0.027992]	[4.38e-05, 0.02263]	[4.2e-05, 0.01890]	[4.03e-05, 0.01618]	[4.38e-05, 0.02185]	[5.55200, 144.8432]	[31.59565, 21011.73]

### 3.4 Moment generating function

The MGF is an important mathematical tool in statistical and probability. It provides an effective means of deriving properties of probability distributions, such as mean, variance, skewness, and flatness. This function helps uniquely characterize and distinguish and is widely used in theoretical analysis and statistical modeling because it simplifies the process of calculating the different moment of distributions.

$$M_{x}\left(t\right)=E\left(e^{\mathrm{tx}}\right)=\int_{-\infty }^{\infty }e^{\mathrm{tx}}f\left(x_{N}\right)\mathrm{dx}$$

After including the moments values derived from our proposed model, we arrived at the following result ^18^:

$$M_{x}\left(t\right)=\sum_{v_{N}=0}^{\infty }\frac{t^{v_{N}}}{v_{N}!}\left[\frac{\Gamma \left(\frac{r-n_{N}}{n_{N}}\right)}{n_{N}k_{N}^{\frac{r-n_{N}}{n_{N}}}}\left[\frac{ϒ}{{\left(2i_{N}\zeta_{N}+2\zeta_{N}+j_{N}+p_{N}\right)}^{\frac{r-n_{N}}{n_{N}}}}-\frac{\Phi }{{\left(2i_{N}\zeta_{N}+2\zeta_{N}+j_{N}+z_{N}\right)}^{\frac{r-n_{N}}{n_{N}}}}\right]\right]$$ (19)

### 3.5 Incomplete moments

Incomplete moments represent a natural extension of the concept of traditional statistical moments, as they are calculated over a specific portion of the distribution domain rather than the entire domain. Their importance lies in their ability to characterize the behavior of a distribution within specific ranges, which making them a powerful tool for analyzing of truncated or skewed data, calculating measures such as mean deviation, studying indices of dispersion and asymmetry, and analyzing inequality curves such as the Lorenz and Bonferroni curves. They also play a key role in economic applications, risk studies, and insurance. The rth incomplete moment of the NNOWIW is mathematically defined as ^19, 20^:

$$\begin{array}{l} M_{r}\left(y\right)=\int_{-\infty }^{y}x_{N}^{r}f\left(x_{N}\right)\mathrm{dx}\\ M_{n}\left(y\right)=\int_{0}^{y}x^{r}\left(ϒx_{N}^{-\left(n_{N}+1\right)}e^{-\left(2i_{N}\zeta_{N}+2\zeta_{N}+j_{N}+p_{N}\right)k_{N}x_{N}^{-n_{N}}}-\Phi x_{N}^{-\left(n_{N}+1\right)}e^{-\left(2i_{N}\zeta_{N}+2\zeta_{N}+j_{N}+z_{N}\right)k_{N}x_{N}^{-n_{N}}}\right)dx_{N}\\ M_{n}\left(y\right)=ϒI_{1}-\Phi I_{2} \end{array}$$

where

$$I_{1}=\int_{0}^{y}x_{N}^{r-\left(n_{N}+1\right)}e^{-\left(2i_{N}\zeta_{N}+2\zeta_{N}+j_{N}+p_{N}\right)k_{N}x_{N}^{-n_{N}}}\mathrm{dx}$$

and

$$I_{2}=\int_{0}^{y}x_{N}^{r-\left(n_{N}+1\right)}e^{-\left(2i_{N}\zeta_{N}+2\zeta_{N}+j_{N}+z_{N}\right)k_{N}x_{N}^{-n_{N}}}\mathrm{dx}$$

For

$I_{1}$ , let

$$\;\begin{array}{c} t=\left(2i_{N}\zeta_{N}+2\zeta_{N}+j_{N}+p_{N}\right)k_{N}x_{N}^{-n_{N}}\\ x=\frac{t^{\frac{1}{n_{N}}}}{k_{N}^{\frac{1}{n_{N}}}{\left(2i_{N}\zeta_{N}+2\zeta_{N}+j_{N}+p_{N}\right)}^{\frac{1}{n_{N}}}}, \end{array}$$

when $x_{N}=0⟹t=0\;$ and if

$$\begin{array}{l} x_{N}=y⟹t=\left(2i_{N}\zeta_{N}+2\zeta_{N}+j_{N}+p_{N}\right)k_{N}y^{-n_{N}}⟹\mathrm{dy}=\frac{t^{\frac{1}{n_{N}}-1}}{n_{N}k_{N}^{\frac{1}{n}}{\left(2i_{N}\zeta_{N}+2\zeta_{N}+j_{N}+p_{N}\right)}^{\frac{1}{n_{N}}}}\mathrm{dt}\\ \;I_{1}=\int_{0}^{\left(2i_{N}\zeta_{N}+2\zeta_{N}+j_{N}+p_{N}\right)k_{N}y^{-n_{N}}}{\left(\frac{t^{\frac{1}{n_{N}}}}{k_{N}^{\frac{1}{n_{N}}}{\left(2i_{N}\zeta_{N}+2\zeta_{N}+j_{N}+p_{N}\right)}^{\frac{1}{n_{N}}}}\right)}^{r-\left(n_{N}+1\right)}e^{-t}\frac{t^{\frac{1}{n_{N}}-1}}{n_{N}k_{N}^{\frac{1}{n}}{\left(2i_{N}\zeta_{N}+2\zeta_{N}+j_{N}+p_{N}\right)}^{\frac{1}{n_{N}}}}\mathrm{dt}\\ \;I_{1}=\frac{\Gamma \left((\frac{r-n_{N}}{n_{N}}),\left(2i_{N}\zeta_{N}+2\zeta_{N}+j_{N}+p_{N}\right)k_{N}y^{-n_{N}}\right)}{n_{N}k_{N}^{\frac{r-n_{N}}{n_{N}}}{\left(2i_{N}\zeta_{N}+2\zeta_{N}+j_{N}+p_{N}\right)}^{\frac{r-n_{N}}{n_{N}}}} \end{array}$$

Following a similar procedure for $I_{2}$ , we arrive at the final form

$$I_{2}=\frac{\Gamma \left((\frac{r-n_{N}}{n_{N}}),\left(2i_{N}\zeta_{N}+2\zeta_{N}+j_{N}+z_{N}\right)k_{N}y^{-n_{N}}\right)}{n_{N}k_{N}^{\frac{r-n_{N}}{n_{N}}}{\left(2i_{N}\zeta_{N}+2\zeta_{N}+j_{N}+z_{N}\right)}^{\frac{r-n_{N}}{n_{N}}}}$$

$$M_{n}\left(y\right)=\frac{ϒ\Gamma \left((\frac{r-n_{N}}{n_{N}}),\left(2i_{N}\zeta_{N}+2\zeta_{N}+j_{N}+p_{N}\right)k_{N}y^{-n_{N}}\right)}{n_{N}k_{N}^{\frac{r-n_{N}}{n_{N}}}{\left(2i_{N}\zeta_{N}+2\zeta_{N}+j_{N}+p_{N}\right)}^{\frac{r-n_{N}}{n_{N}}}}-\frac{\Phi \Gamma \left((\frac{r-n_{N}}{n_{N}}),\left(2i_{N}\zeta_{N}+2\zeta_{N}+j_{N}+z_{N}\right)k_{N}y^{-n_{N}}\right)}{n_{N}k_{N}^{\frac{r-n_{N}}{n_{N}}}{\left(2i_{N}\zeta_{N}+2\zeta_{N}+j_{N}+z_{N}\right)}^{\frac{r-n_{N}}{n_{N}}}}$$ (20)

### 3.6 Lorenz and Bonferroni curves

The Lorenz curve is a traditional graphical tool used to represent the cumulative distribution of income or wealth and compare this distribution to an ideal line of equality, helping to measure the degree of inequality. The Bonferroni curve an extension and improvement of the Lorenz curve, provides a more accurate analysis by incorporation additional measures of inequality, making it more sensitive to changes in lower-income groups. Thus, the Bonferroni curve provides deeper insights than the Lorenz curve, especially when studying economic inequality. The Lorenz $\left(L_{F}\left(y\right)\right)$ and Bonferroni $\left(B_{F}\left(y\right)\right)$ curves are mathematically defined by ^21– 23^:

$$L_{F}\left(y\right)=\frac{1}{\mu }\int_{0}^{y}x_{N}\;f\left(x_{N}\right)d,\;B_{F}\left(y\right)=\frac{L_{F}\left(y\right)}{F\left(x_{N}\right)}$$

Accordingly, the Lorenz curve $L_{F}\left(y\right)$ and the Bonferroni curve $B_{F}\left(y\right)$ are derived, respectively:

$$L_{F}\left(y\right)=\frac{ϒ}{\mu n_{N}}{\left(\left(2i_{N}\zeta_{N}+2\zeta_{N}+j_{N}+p_{N}\right)k_{N}\right)}^{\frac{1}{n_{N}}-1}Τ\left(\left(2i_{N}\zeta_{N}+2\zeta_{N}+j_{N}+p_{N}\right)k_{N}y^{-n_{N}}\right)-\frac{\Phi }{\mu n_{N}}{\left(\left(2i_{N}\zeta_{N}+2\zeta_{N}+j_{N}+z_{N}\right)k_{N}\right)}^{\frac{1}{n_{N}}-1}Τ\left(1-\frac{1}{n_{N}},\left(2i_{N}\zeta_{N}+2\zeta_{N}+j_{N}+z_{N}\right)k_{N}y^{-n_{N}}\right)$$ (21)

$$B_{F}\left(y\right)=\frac{\left[\begin{array}{l} \frac{ϒ}{\mu n_{N}}{\left(\left(2i_{N}\zeta_{N}+2\zeta_{N}+j_{N}+p_{N}\right)k_{N}\right)}^{\frac{1}{n_{N}}-1}Τ\left(\left(2i_{N}\zeta_{N}+2\zeta_{N}+j_{N}+p_{N}\right)k_{N}y^{-n_{N}}\right)\\ -\frac{\Phi }{\mu n_{N}}{\left(\left(2i_{N}\zeta_{N}+2\zeta_{N}+j_{N}+z_{N}\right)k_{N}\right)}^{\frac{1}{n_{N}}-1}Τ\left(1-\frac{1}{n_{N}},\left(2i_{N}\zeta_{N}+2\zeta_{N}+j_{N}+z_{N}\right)k_{N}y^{-n_{N}}\right) \end{array}\right]}{1-e^{\left(-\eta_{N}{\left[-e^{-k_{N}x_{N}^{-n_{N}}}.\log\left(1-e^{-k_{N}x_{N}^{-n_{N}}}\right)\right]}^{\zeta_{N}}\right)}}$$ (22)

### 3.7 Probability-weighted moments

If random variable X follows the NNOWIW distribution, the Probability Weighted Moments (PWM) can be calculated using the following formula ^24^:

$$E\left(X^{p}{\left(F\left(x\right)\right)}^{q}\right)=\int_{0}^{\infty }x_{N}^{p}f\left(x_{N}\right){\left(F\left(x_{N}\right)\right)}^{q}dx_{N}\;p=1,2,3,\ldots \;,\;q=0,1,2,\ldots \;$$

By using the NCDF and NPDF into the expression $f\left(x_{N}\right){\left(F\left(x_{N}\right)\right)}^{q}$ , a series expansion can be used to simplify the algebraic formulation, resulting in the following linear representation ^24^:

$$E\left(X^{p}{\left(F\left(x\right)\right)}^{q}\right)=\frac{\Gamma \left(\frac{r-n_{N}}{n_{N}}\right)}{n_{N}{k_{N}}^{\frac{r-n_{N}}{n_{N}}}}\left[\frac{\Omega }{{\left(j_{N}\zeta_{N}+\zeta_{N}+s\_N+1\right)}^{\frac{r-n_{N}}{n_{N}}}}-\frac{\Psi }{{\left(j_{N}\zeta_{N}+\zeta_{n}+r_{n}\right)}^{\frac{r-n}{n}}}\right]$$ (23)

Where

$$\Omega =\sum_{i_{N}=j_{N}=p_{N}=s_{N}=0}^{\infty }\frac{{\left(-1\right)}^{i_{N}+2j_{N}+\zeta_{N}-1+p_{N}+s_{N}}\left(\eta_{N}+i_{N}\eta_{N}\right)}{j!}\left(\begin{array}{c} q\\ i_{N} \end{array}\right)d_{j_{N}\zeta_{N}+\zeta_{N}-1,p_{N}}\eta_{N}\zeta_{N}k_{N}n_{N}$$

$$\Psi =\sum_{i_{N}=j_{N}=p_{N}=r_{N}=0}^{\infty }\frac{{\left(-1\right)}^{i_{N}+2j_{N}+\zeta_{N}-1+p_{N}+R_{N}}\left(\eta_{N}+i_{N}\eta_{N}\right)}{j!}d_{j_{N}\zeta_{N}+\zeta_{N}-1,p_{N}}\eta_{N}\zeta_{N}k_{N}n_{N}$$

### 3.8 Characteristic function

The characteristic function is a fundamental tool in probability and statistics, used to accurately describe the probability distribution of a random variable. It is defined as the expected value of the exponential complex number $e^{\mathrm{itx}}$ , where i is the imaginary unit. This function is distinguished by its ability to derive moments and understand the behavior of a sum of random variables, in addition to its persistence even in cases where the moment generating function (MGF) is not present. The Characteristic function can be calculated using the following formula ^25^:

$$Q_{x}\left(t\right)=E\left(e^{\mathrm{itx}}\right)=\int_{0}^{\infty }e^{\mathrm{itx}}f\left(x_{N}\right)\;\mathrm{dx}$$

Using exponential expansion for above equation, to get a form:

$$Q_{x}\left(t\right)=E\left(e^{\mathrm{itx}}\right)=\sum_{q_{N}=0}^{\infty }\frac{{\left(\mathrm{it}\right)}^{q_{N}}}{q_{N}!}\left[\mu_{r}\right]$$

$$Q_{x}\left(t\right)=\sum_{q_{N}=0}^{\infty }\frac{{\left(\mathrm{it}\right)}^{q_{N}}}{q_{N}!}\left[\frac{\Gamma \left(\frac{r-n_{N}}{n_{N}}\right)}{n_{N}k_{N}^{\frac{r-n_{N}}{n_{N}}}}\left[\frac{ϒ}{{\left(2i_{N}\zeta_{N}+2\zeta_{N}+j_{N}+p_{N}\right)}^{\frac{r-n_{N}}{n_{N}}}}-\frac{\Phi }{{\left(2i_{N}\zeta_{N}+2\zeta_{N}+j_{N}+z_{N}\right)}^{\frac{r-n_{N}}{n_{N}}}}\right]\right]$$ (24)

### 3.9 Entropy measures

Entropy is a fundamental concept in information theory and statistics, used to measure the level of uncertainty or randomness in a probability distribution. In the context of continuous lifetime distributions, entropy provides deep insights into the internal properties and variance of the data. This paper relies on three different entropy measures to characterize the proposed distribution. The Rényi entropy measure for NNOWIW distribution, denoted by $I_{R}\left(c\right)$ , is derived using chain expansions, yielding the following expression ^26, 27^:

$$\begin{array}{c} I_{R}\left(c\right)=\frac{1}{1-c}\log\int_{0}^{\infty }f{\left(x_{N}\right)}^{c}\mathrm{dx}\\ I_{R}\left(c\right)=\frac{1}{1-c}\log\left[\int_{0}^{\infty }{\left(ϒx_{N}^{-\left(n_{N}+1\right)}e^{-\left(2i_{N}\zeta_{N}+2\zeta_{N}+j_{N}+p_{N}\right)k_{N}x_{N}^{-n_{N}}}-\Phi x_{N}^{-\left(n_{N}+1\right)}e^{-\left(2i_{N}\zeta_{N}+2\zeta_{N}+j_{N}+z_{N}\right)k_{N}x_{N}^{-n_{N}}}\right)}^{c}\mathrm{dx}\right] \end{array}$$

Using the Binomial Series

$$I_{R}\left(c\right)=\frac{1}{1-c}\log\left[\int_{0}^{\infty }\sum_{s_{N}=0}^{c}{\left(-1\right)}^{s_{N}}\left(\begin{array}{c} c\\ \;s_{N}\; \end{array}\right){ϒ}^{c-s_{N}}\Phi^{s_{N}}x^{-c\left(n_{N}+1\right)}\;e^{-\left(2i_{N}\zeta_{N}c+2\mathrm{\zeta c}+j_{N}c+p_{N}c-s_{N}p_{N}+s_{N}z_{N}\right)k_{N}x_{N}^{-n_{N}}}dx_{N}\right]$$

Let

$$\begin{array}{l} \;u=\left(2i_{N}\zeta_{N}c+2\mathrm{\zeta c}+j_{N}c+p_{N}c-s_{N}p_{N}+s_{N}z_{N}\right)k_{N}x_{N}^{-n_{N}}⟹\\ \;x=\frac{u^{\frac{1}{n_{N}}}}{k^{\frac{1}{n_{N}}}{\left(2i_{N}\zeta_{N}c+2\mathrm{\zeta c}+j_{N}c+p_{N}c-s_{N}p_{N}+s_{N}z_{N}\right)}^{\frac{1}{n_{N}}}}\\ \;dx_{N}=\frac{u^{\frac{1}{n_{N}}-1}}{n_{N}k_{N}^{\frac{1}{n_{N}}}{\left(2i_{N}\zeta_{N}c+2\mathrm{\zeta c}+j_{N}c+p_{N}c-s_{N}p_{N}+s_{N}z_{N}\right)}^{\frac{1}{n_{N}}}}\mathrm{du}\\ I_{R}{\left(c\right)}_{\text{NNOWIW}}=\frac{1}{1-c}\log\left[\int_{0}^{\infty }\sum_{s_{N}=0}^{c}{\left(-1\right)}^{s_{N}}\left(\begin{array}{c} c\\ s_{N} \end{array}\right){ϒ}^{c-s_{N}}\Phi^{s\_N}{\left(\frac{u^{\frac{1}{n_{N}}}}{k^{\frac{1}{n_{N}}}{\left(2i_{N}\zeta_{N}c+2\mathrm{\zeta c}+j_{N}c+p_{N}c-s_{N}p_{N}+s_{N}z_{N}\right)}^{\frac{1}{n_{N}}}}\right)}^{-c\left(n_{N}+1\right)}\right]\\ \;e^{-u}\frac{u^{\frac{1}{n_{N}}-1}}{n_{N}k_{N}^{\frac{1}{n_{N}}}{\left(2i_{N}\zeta_{N}c+2\mathrm{\zeta c}+j_{N}c+p_{N}c-s_{N}p_{N}+s_{N}z_{N}\right)}^{\frac{1}{n_{N}}}}\mathrm{du} \end{array}$$

Put

$$\begin{array}{l} \varpi =\sum_{s_{N}=0}^{c}{\left(-1\right)}^{s_{N}}\left(\begin{array}{c} c\\ s_{N} \end{array}\right){ϒ}^{c-s_{N}}\Phi^{s\_N}\frac{1}{k^{\frac{-c\left(n_{N}+1\right)+1}{n_{N}}}{\left(2i_{N}\zeta_{N}c+2\mathrm{\zeta c}+j_{N}c+p_{N}c-s_{N}p_{N}+s_{N}z_{N}\right)}^{\frac{-c\left(n_{N}+1\right)+1}{n_{N}}}}\\ I_{R}{\left(c\right)}_{\text{NNOWIW}}=\frac{1}{1-c}\log\left[\varpi \int_{0}^{\infty }u^{\frac{-c\left(n_{N}+1\right)}{n_{N}}+\frac{1}{n_{N}}-1}e^{-u}\mathrm{du}\right] \end{array}$$

$$I_{R}{\left(c\right)}_{\text{NNOWIW}}=\frac{1}{1-c}\log\left[\varpi .\Gamma \left(\frac{1-c\left(n_{N}+1\right)}{n_{N}}\right)\right]$$ (25)

Where

$$\varpi =\sum_{s_{N}=0}^{c}{\left(-1\right)}^{s_{N}}\left(\begin{array}{c} c\\ s_{N} \end{array}\right){ϒ}^{c-s_{N}}\Phi^{s\_N}\frac{1}{k^{\frac{-c\left(n_{N}+1\right)}{n_{N}}+\frac{1}{n_{N}}}{\left(2i_{N}\zeta_{N}c+2\mathrm{\zeta c}+j_{N}c+p_{N}c-s_{N}p_{N}+s_{N}z_{N}\right)}^{\frac{-c\left(n_{N}+1\right)}{n_{N}}+\frac{1}{n_{N}}}}$$

The mathematical representation of the Havrda and Charvát entropy is given by ^28^:

$$I_{\mathrm{HC}}\left(c\right)=\frac{1}{2^{1-c}-c}\left({\left(\int_{0}^{\infty }f^{c}\left(x\right)\mathrm{dx})\right)}^{\frac{1}{c}}-1\right)\;,\;c>0,\;c\neq 1$$

It is noted that the integration used here is very similar to that used in calculating the Renny entropy. Therefore, the Havrda and Charvat entropies for the NNOWIW distribution can be represented as follows:

$$I_{\mathrm{HC}}\left(c\right)=\frac{1}{2^{1-c}-c}\left({\left(\varpi .\Gamma \left(\frac{1-c\left(n+1\right)}{n}\right)\right)}^{\frac{1}{c}}-1\right)\;,\;c>0,\;c\neq 1$$ (26)

The mathematical representation of the Tsallis’s Entropy is given by ^29^:

$$T_{c}=\frac{1}{1-c}\left(1-\int_{0}^{\infty }f^{c}\left(x\right)\mathrm{dx}\right)\;,\;c>0,\;c\neq 1$$

For the NNOWIW random variable, the Tsallis entropy is given by:

$$T_{c}=\frac{1}{1-c}\left(1-\varpi .\Gamma \left(\frac{1-c\left(n+1\right)}{n}\right)\right)\;,\;c>0,\;c\neq 1$$ (27)

The mathematical representation of the Arimoto Entropy is given by ^30^:

$$A_{c}=\frac{c}{1-c}\left({\left(\int_{-\infty }^{\infty }f^{c}\left(x\right)\mathrm{dx})\right)}^{\frac{1}{c}}-1\right)\;,\;c>0,\;c\neq 1$$

for the NNOWIW random variable, the Arimoto entropy is given by:

$$A_{c}=\frac{c}{1-c}\left({\left(\varpi .\Gamma \left(\frac{1-c\left(n+1\right)}{n}\right)\right)}^{\frac{1}{c}}-1\right)\;,\;c>0,\;c\neq 1$$ (28)

## 4. Estimation methods

In this section, we review some of the most prominent statistical estimation methods, including the maximum likelihood method (MLE), the least square method (LSE), and the weighted lest squares method (WLSE). These methods aim to find optimal values for parameters, either by maximizing the likelihood function or minimizing the error functions, which contributes to improving the accuracy of the statistical model and the interpretation of its results.

### 4.1 Maximum likelihood method

In this paragraph, we review one of the most common estimation methods, the MLE which is used to estimate the parameters $\eta_{N},\zeta_{N},k_{N},n_{N}$ for the NNOWIW distribution. Suppose that $X_{1},X_{2},\ldots ,X_{n}$ represents a random sample from the NNOWIW distribution, and that $x_{1},x_{2},\ldots ,x_{n}$ represents the observed values of this sample. In this case, the log-likelihood function is given by ^31– 33^:

$$L\left(\theta_{N},{x_{N}}_{i}\right)=\prod_{i=1}^{m}\eta_{N}\zeta_{N}k_{N}n_{N}x_{N}^{-\left(n+1\right)}e^{-k_{N}x_{N}^{-n_{N}}}\left[\frac{e^{-k_{N}x_{N_{i}}^{-n_{N}}}}{1-e^{-k_{N}x_{N_{i}}^{-n_{N}}}}-\log\left(1-e^{-k_{N}x_{N_{i}}^{-n_{N}}}\right)\right]\times {\left[-e^{-k_{N}x_{N_{i}}^{-n_{N}}}.\log\left(1-e^{-k_{N}x_{N_{i}}^{-n_{N}}}\right)\right]}^{\zeta_{N}-1}e^{\left(-\eta_{N}{\left[-e^{-k_{N}x_{N_{i}}^{-n_{N}}}.\log\left(1-e^{-k_{N}x_{N_{i}}^{-n_{N}}}\right)\right]}^{\zeta_{N}}\right)}$$

we compute the log-likelihood:

$$L=m\text{log}\left(\eta_{N}\right)+m\text{log}\left(\zeta_{N}\right)+m\text{log}\left(k_{N}\right)+m\text{log}\left(n_{N}\right)-\left(n_{N}-1\right)\sum_{i=1}^{m}\log\left({x_{N}}_{i}\right)-\sum_{i=1}^{m}k_{N}x_{N_{i}}^{-n_{N}}+\sum_{i=1}^{m}\log\left[\frac{e^{-k_{N}x_{N_{i}}^{-n_{N}}}}{1-e^{-k_{N}x_{N_{i}}^{-n_{N}}}}-\log\left(1-e^{-k_{N}x_{N_{i}}^{-n_{N}}}\right)\right]+\left(\zeta_{N}-1\right)\sum_{i=1}^{m}\log\left[-e^{-k_{N}x_{N_{i}}^{-n_{N}}}.\log\left(1-e^{-k_{N}x_{N_{i}}^{-n_{N}}}\right)\right]-\eta_{N}\sum_{i=1}^{m}{\left[-e^{-k_{N}x_{N_{i}}^{-n_{N}}}.\log\left(1-e^{-k_{N}x_{N_{i}}^{-n_{N}}}\right)\right]}^{\zeta_{N}}$$ (29)

### 4.2 Least squares method

This paragraph discusses the use of the LSE method to estimate the parameters of the NNOWIW distribution. This method relies on finding estimated values for the parameters by minimizing the square error function between the theoretical and experimental values. LSE are defined as the values that minimize the function given in Equation (30), thus ensuring that the difference between the theoretical CDF and its experimental counterpart is minimized. ^34, 35^

$$\varphi \left(\theta_{N}\right)=\sum_{i=1}^{m}{\left[1-e^{\left(-\eta {\left[-e^{-kx^{-n}}.\log\left(1-e^{-kx^{-n}}\right)\right]}^{\zeta }\right)}-\frac{1}{n+1}\right]}^{2}$$ (30)

### 4.3 Weighted least squares method

This paragraph presents $\mathrm{WLS}E_{s}$ for the parameters $\eta_{N},\zeta_{N},k_{N},n_{N}$ of the NNOWIW distribution. These estimators are obtained by minimizing the function in Equation (31), ensuring that the differences between the theoretical and experimental values are minimized while giving greater weight to more accurate observations. ^35, 36^

$$W\left(\theta_{N}\right)=\sum_{i=1}^{m}\frac{{\left(n+1\right)}^{2}\left(n+2\right)}{i\left(n-i+1\right)}{\left[1-e^{\left(-\eta {\left[-e^{-kx^{-n}}.\log\left(1-e^{-kx^{-n}}\right)\right]}^{\zeta }\right)}-\frac{i}{n+1}\right]}^{2}$$ (31)

In the Neutrosophic framework, the parameters $\eta_{N},\zeta_{N},k_{N},n_{N}$ are considered as interval-valued rather than single-point estimates, i.e, $\theta_{N}\in \left[\theta_{L},\theta_{U}\right]$ . For MLE, the likelihood function is maximized within these bounds, yielding estimates expressed as parameter intervals. Similarly, in LSE and WLSE, the minimization of the error functions is performed while accounting for the interval nature of the parameters. This adjustment allows the estimation methods to explicitly reflect the inherent indeterminacy and partial information in the data, which would not be possible under the classical framework.

## 5. Monte carlo simulation for NMWIW distribution

Monte Carlo simulation is a powerful statistical tool for studying the performance of parameter estimators in complex models. It relies on generating multiple random samples to accurately estimate statistical indicators. In this section, we apply Monte Carlo simulation to the NNOWIW distribution using three estimations: MLE, LSE, WLSE. The effectiveness of these methods is evaluated using three key metrics: MSE, RMSE, Bais, across 1000 iteration for different sample size (50, 100, 150, and 200).

Table 3 provides a summary of the simulation results, showing the three performance measures for each sample size, allowing for a practical assessment of the effectiveness of the estimation methods used.

**Table 3.** Monte Carlo simulations conducted for the NNOWIW.

$\eta_{N}=\left[2.9,3.9\;\right],\;\zeta_{N}=\left[2.3,3.3\;\right],\;k_{N}=\left[2.7,\;3.7\;\right],\;n_{N}=\left[2.9,3.9\;\right]$					
N	Est.	Ess. Par.	MLE	LSE	WLSE
50	Mean	$\hat{\eta_{N}}$	[5.01658, 5.28062]	[3.04048, 3.97395]	[3.23027, 4.22279]
		$\hat{\zeta_{N}}$	[3.76140, 3.88307]	[2.25692, 3.24020]	[2.45622, 3.22748]
		$\hat{k_{N}}$	[2.51916, 4.39999]	[3.08007, 3.99232]	[3.05685, 4.22692]
		$\hat{n_{N}}$	[2.50111, 3.92786]	[3.06710, 3.99681]	[3.03765, 4.04226]
	MSE	$\hat{\eta_{N}}$	[24.33201, 31.07039]	[2.72677, 3.15312]	[4.26974, 5.09414]
		$\hat{\zeta_{N}}$	[2.23281, 5.08498]	[0.32050, 0.49037]	[0.44506, 0.59960]
		$\hat{k_{N}}$	[6.73629, 10.68197]	[1.24320, 1.63249]	[2.93147, 3.08101]
		$\hat{n_{N}}$	[1.55346, 1.87444]	[0.31983, 0.68382]	[0.65505, 1.06296]
	RMSE	$\hat{\eta_{N}}$	[4.93275, 5.57408]	[1.65129, 1.77570]	[2.06634, 2.25702]
		$\hat{\zeta_{N}}$	[1.49426, 2.25499]	[0.56613, 0.70027]	[0.66713, 0.77434]
		$\hat{k_{N}}$	[2.59544, 3.26833]	[1.11499, 1.27769]	[1.71215, 1.75528]
		$\hat{n_{N}}$	[1.24638, 1.36910]	[0.56553, 0.82693]	[0.80935, 1.03100]
	Bias	$\hat{\eta_{N}}$	[1.38062, 2.11658]	[0.07395, 0.14048]	[0.32279, 0.33027]
		$\hat{\zeta_{N}}$	[0.58307, 1.46140]	[0.04308, 0.05980]	[0.07252, 0.15622]
		$\hat{k_{N}}$	[0.18085, 0.69999]	[0.29232, 0.38007]	[0.35685, 0.52692]
		$\hat{n_{N}}$	[0.02786, 0.39889]	[0.09681, 0.16710]	[0.13765, 0.14226]
100	Mean	$\hat{\eta_{N}}$	[4.37084, 5.23464]	[3.17529, 4.02769]	[3.58183, 4.43313]
		$\hat{\zeta_{N}}$	[3.29612, 3.74326]	[2.34171, 3.20355]	[2.55049, 3.28890]
		$\hat{k_{N}}$	[2.45184, 4.35710]	[3.05476, 3.98946]	[2.83991, 4.09967]
		$\hat{n_{N}}$	[2.56868, 3.93777]	[3.07363, 4.00434]	[2.90707, 3.99445]
	MSE	$\hat{\eta_{N}}$	[16.64141, 21.18204]	[3.13994, 3.54256]	[5.37979, 5.61810]
		$\hat{\zeta_{N}}$	[1.77969, 2.93891]	[0.29048, 0.35044]	[0.37845, 0.59093]
		$\hat{k_{N}}$	[2.74310, 9.08399]	[1.24016, 1.76450]	[1.96371, 2.31814]
		$\hat{n_{N}}$	[1.02224, 1.33899]	[0.35861, 0.73516]	[0.58162, 0.78987]
	RMSE	$\hat{\eta_{N}}$	[4.07939, 4.60240]	[1.77199, 1.88217]	[2.31944, 2.37025]
		$\hat{\zeta_{N}}$	[1.33405, 1.71432]	[0.53897, 0.59198]	[0.61518, 0.76872]
		$\hat{k_{N}}$	[1.65623, 3.01397]	[1.11363, 1.32834]	[1.40132, 1.52255]
		$\hat{n_{N}}$	[1.01106, 1.15715]	[0.59884, 0.85741]	[0.76264, 0.88875]
	Bias	$\hat{\eta_{N}}$	[1.33464, 1.47084]	[0.12769, 0.27529]	[0.53313, 0.68183]
		$\hat{\zeta_{N}}$	[0.44326, 0.99612]	[0.04171, 0.09645]	[0.01110, 0.25049]
		$\hat{k_{N}}$	[0.24816, 0.65710]	[0.28946, 0.35476]	[0.13991, 0.39967]
		$\hat{n_{N}}$	[0.03777, 0.33132]	[0.10434, 0.17363]	[0.00708, 0.09445]
150	Mean	$\hat{\eta_{N}}$	[3.87006, 5.34547]	[3.33316, 4.07315]	[3.43484, 4.23583]
		$\hat{\zeta_{N}}$	[3.01773, 3.68578]	[2.36775, 3.21317]	[2.50387, 3.28096]
		$\hat{k_{N}}$	[2.41915, 4.24814]	[2.95700, 4.10125]	[2.91116, 4.19146]
		$\hat{n_{N}}$	[2.62497, 3.90406]	[2.98450, 4.03178]	[2.93553, 4.07096]
	MSE	$\hat{\eta_{N}}$	[7.06217, 28.14989]	[2.93271, 3.07076]	[4.31844, 5.95674]
		$\hat{\zeta_{N}}$	[1.54360, 1.71964]	[0.24809, 0.26853]	[0.35666, 0.45342]
		$\hat{k_{N}}$	[1.75399, 7.94597]	[1.38113, 1.62891]	[2.13059, 2.56299]
		$\hat{n_{N}}$	[0.68099, 1.18606]	[0.41767, 0.56877]	[0.67345, 0.71091]
	RMSE	$\hat{\eta_{N}}$	[2.65747, 5.30565]	[1.71252, 1.75236]	[2.07809, 2.44064]
		$\hat{\zeta_{N}}$	[1.24242, 1.31135]	[0.49809, 0.51820]	[0.59721, 0.67336]
		$\hat{k_{N}}$	[1.32438, 2.81886]	[1.17522, 1.27629]	[1.45965, 1.60093]
		$\hat{n_{N}}$	[0.82522, 1.08906]	[0.64627, 0.75417]	[0.82064, 0.84316]
	Bias	$\hat{\eta_{N}}$	[0.97006, 1.44547]	[0.17315, 0.43316]	[0.33583, 0.53484]
		$\hat{\zeta_{N}}$	[0.38578, 0.71773]	[0.06775, 0.08683]	[0.01904, 0.20387]
		$\hat{k_{N}}$	[0.28085, 0.54814]	[0.25700, 0.40125]	[0.21116, 0.49146]
		$\hat{n_{N}}$	[0.00406, 0.27503]	[0.08450, 0.13178]	[0.03553, 0.17096]
200	Mean	$\hat{\eta_{N}}$	[4.23646, 5.08184]	[3.19814, 4.05351]	[3.41139, 4.30858]
		$\hat{\zeta_{N}}$	[2.84721, 3.58236]	[2.37044, 3.26090]	[2.51590, 3.36403]
		$\hat{k_{N}}$	[2.52360, 4.28501]	[2.98316, 4.00045]	[2.75885, 3.96740]
		$\hat{n_{N}}$	[2.65832, 3.94285]	[3.02275, 4.00592]	[2.86904, 3.97495]
	MSE	$\hat{\eta_{N}}$	[12.99904, 16.95849]	[2.77691, 2.80402]	[3.83196, 5.67453]
		$\hat{\zeta_{N}}$	[1.18934, 1.26479]	[0.19534, 0.28128]	[0.30394, 0.41848]
		$\hat{k_{N}}$	[1.66484, 6.87412]	[1.13695, 1.43052]	[1.26359, 1.92884]
		$\hat{n_{N}}$	[0.64359, 1.01041]	[0.29588, 0.60924]	[0.53436, 0.56316]
	RMSE	$\hat{\eta_{N}}$	[3.60542, 4.11807]	[1.66641, 1.67452]	[1.95754, 2.38213]
		$\hat{\zeta_{N}}$	[1.09057, 1.12463]	[0.44198, 0.53036]	[0.55130, 0.64690]
		$\hat{k_{N}}$	[1.29029, 2.62185]	[1.06628, 1.19604]	[1.12410, 1.38883]
		$\hat{n_{N}}$	[0.80224, 1.00519]	[0.54395, 0.78054]	[0.73100, 0.75044]
	Bias	$\hat{\eta_{N}}$	[1.18184, 1.33646]	[0.15351, 0.29814]	[0.40858, 0.51139]
		$\hat{\zeta_{N}}$	[0.28236, 0.54721]	[0.03910, 0.07044]	[0.06403, 0.21590]
		$\hat{k_{N}}$	[0.17640, 0.58501]	[0.28316, 0.30045]	[0.05885, 0.26740]
		$\hat{n_{N}}$	[0.04285, 0.24168]	[0.10592, 0.12275]	[0.03096, 0.07495]

The results of Table 3 show that increasing the sample size improves that estimation efficiency by reducing the MSE, RMSE, and Bias values, reflecting higher accuracy in estimating the NNOWIW distribution parameters. The MLE method provides the best results with large samples, while the LSE and WLSE methods perform well in some cases, especially with small samples or skewed data. It is worth noting that some fluctuations appear due to the randomness of Monte Carlo simulations, but the overall trend confirms the improvement with large samples.

## 6. Application

To demonstrate the effectiveness of the NNOWIW distribution in achieving an accurate fit to the data, we present a practical example using two real-world data sets. This example aims to illustrate the advantages of the NNOWIW distribution and its fit to the data. Table 4 presents a comparative analysis between the NNOWIW distribution and several other distributions using the validated data.

**Table 4.** CDF functions for comparative distributions.

Distribution	CDF
Neutrosophic New Odd Weibull Inverse Weibull	$1-e^{\left(-\eta_{N}{\left[-e^{-k_{N}x_{N}^{-n_{N}}}.\text{log}\left(1-e^{-k_{N}x_{N}^{-n_{N}}}\right)\right]}^{\zeta_{N}}\right)}$
Neutrosophic beta inverse Weibull	$\mathrm{\rho B}\left(e^{-k_{N}x_{N}^{-n_{N}}}\;,\eta_{N},\zeta_{N}\right)$
Neutrosophic Kumaraswamy inverse Weibull	$1-{\left(1-{\left(e^{-k_{N}x_{N}^{-n_{N}}}\right)}^{\eta_{N}}\right)}^{\zeta_{N}}$
Neutrosophic Exponeted generalized inverse Weibull	${\left(1-{\left(1-e^{-k_{N}x_{N}^{-n_{N}}}\right)}^{\eta_{N}}\right)}^{\zeta_{N}}$
Neutrosophic log-Gamma inverse Weibull	$1-\rho \;\text{gamma}\left(-\zeta_{N}.\text{log}\left(1-e^{-k_{N}x_{N}^{-n_{N}}}\right),\eta_{N}\right)\;$
Neutrosophic Gompertz inverse Weibull	$1-e^{\frac{\eta_{N}}{\zeta_{N}}\left\{1-{\left(1-e^{-k_{N}x_{N}^{-n_{N}}}\right)}^{-\zeta_{N}}\right\}}$
Neutrosophic inverse Weibull	$e^{-k_{N}x_{N}^{-n_{N}}}$

These distributions were chosen due to their similar mathematical structure and ability to handle uncertain data. All of these distributions belong to extended or modified families of the Inverse Weibull distribution, making them direct competitors to the NNOWIW distribution. They are characterized by their ability to represent ambiguous or time-interval data, which is the primary goal of the NNOWIW model. Four informative criteria were used ^37– 42^:

$$\begin{array}{l} \mathrm{AIC}=-2\sum_{j=1}^{T}\log f_{m}\left(x_{j}/{\hat{\theta }}_{m}\right)+2k\\ \text{CAIC}=\mathrm{AIC}+\frac{2k\left(k+1\right)}{n-k-1}\\ \begin{array}{c} \text{HQIC}=2k\;\ln\left[\ln\left(n\right)\right]-2l\left(\hat{\theta }\right)\\ \mathrm{BIC}=-2l\left(\hat{\theta }\right)+k\;\log\left(n\right) \end{array} \end{array}$$

In addition to four statistical measures to assess accuracy ^43– 44^:

$$\begin{array}{l} K-S=\underset{n}{\sup}\left|F_{n}\left(x\right)-F\left(x\right)\right|\\ A=\sqrt{-n-\frac{1}{n}\sum_{i=1}^{n}\left(2i-1\right)H}\\ H=\left[\ln F\left(x_{i}\right)+\left(1-\ln F\left(x_{n+1-i}\right)\right)\right]\\ W=\sqrt{\frac{1}{12n}+\sum_{i=1}^{n}{\left(F\left(x_{i}\right)-\frac{2i-1}{2n}\right)}^{2}}\\ p‐\text{value}=P\left(T\geq T_{\mathrm{obs}}|H_{0}\right) \end{array}$$

### 6.1 Data set

The data represent the lifetime of batteries. The lifetime in 100 hours of 23 batteries is given as. ^45^

Table 5 shows that the number of observations (23) is sufficient for a preliminary statistical analysis, despite the relatively medium sample size. The mean (mean) ranges between [20.59, 28.19], which represents approximately the expected value for the variable under study. However, this value may be affected by outliers, especially in the presence of skewness. The standard deviation (SD) of [15.93, 21.46] indicates a moderate amount of variance among the values. The skewness coefficient (SK) of [1.77, 1.71] indicates a clear positive skew, with most values concentrated on the lower end of the data, with the tail extending to the higher values. This is a common pattern in life expectancy data or similar data. Kurtness (KU) of [3.32, 4.48] indicates that the distribution is steeper than the normal distribution (which has skewness = 3), meaning there is greater centering around the mean values compared to the normal distribution.

**Table 5.** Descriptive statistics of the data.

Var	N	Mean	SD	Median	Trimmed	Mad	Min	Max	Range	SK	KU	Se
	23	[20.59, 28.19]	[15.93, 21.46]	[17.05, 23.45]	[18.1, 24.89]	[8.08, 11.1]	[2.9, 3.99]	[73.48, 98.04]	[70.58, 94.05]	[1.77, 1.71]	[3.04, 2.71]	[3.32, 4.48]

Table 6 shows a summary of the criteria for selecting the models used in comparing the distributions, while Table 7 shows the values resulting from the statistical tests. Table 8 includes the estimated values of the parameters of each distribution under study.

**Table 6.** Distribution evaluation criteria results.

Dist.	-Log	AIC	CAIC	BIC	HQIC
NNOWIW	[88.62533, 95.80281]	[185.2507, 199.605]	[187.4729, 201.827]	[189.7926, 204.147]	[186.393, 200.7479]
NBeIW	[89.6844, 97.2003]	[187.3769, 202.431]	[189.5991, 204.653]	[191.9189, 206.973]	[188.5192, 203.573]
NKuIW	[88.9879, 96.65249]	[185.9769, 201.317]	[188.1991, 203.539]	[190.5189, 205.859]	[187.1192, 202.459]
NEGIW	[90.02595, 97.52312]	[188.0574, 203.060]	[190.2796, 205.282]	[192.5994, 207.602]	[189.1997, 204.203]
NLGamIW	[89.81861, 96.8195]	[187.6456, 201.640]	[189.8679, 203.863]	[192.1876, 206.182]	[188.7879, 202.783]
NGoIW	[89.71942, 96.95312]	[187.4389, 201.906]	[189.6611, 204.128]	[191.9808, 206.448]	[188.5811, 203.048]
NIW	[91.39956, 98.65839]	[186.7991, 201.316]	[187.3991, 201.916]	[189.0701, 203.587]	[187.3703, 201.887]

**Table 7.** Value of the statistical measures.

Dist.	W	A	K-S	p-value
NNOWIW	[0.06499, 0.06663]	[0.35799, 0.36585]	[0.13708, 0.13841]	[0.71967, 0.73024]
NBeIW	[0.10091, 0.10208]	[0.55900, 0.56697]	[0.17874, 0.19775]	[0.28962, 0.40660]
NKuIW	[0.07621, 0.08998]	[0.42074, 0.49959]	[0.14289, 0.17939]	[0.40218, 0.68340]
NEGIW	[0.11057, 0.11098]	[0.61405, 0.61767]	[0.18435, 0.19613]	[0.29854, 0.36938]
NLGamIW	[7.08964, 7.13061]	[45.57148, 45.64410]	[0.99728, 0.99730]	[4.440892e-16, 4.440892e-16]
NGoIW	[0.10245, 0.10330]	[0.57442, 0.58167]	[0.14507, 0.14701]	[0.64978, 0.66569]
NIW	[0.15295, 0.15333]	[0.85354, 0.85425]	[0.20822, 0.20970]	[0.22945, 0.23637]

**Table 8.** Estimator value interval for parameters by MLE.

Dist.	${\hat{k}}_{N}$	${\hat{\hat{\zeta }}}_{N}$	${\hat{\eta }}_{N}$	${\hat{\zeta }}_{N}$
NNOWIW	[0.48626, 2.23568]	[2.31023, 2.95356]	[1.57355, 2.67725]	[0.55261, 0.63860]
NBeIW	[4.41993, 4.68428]	[2.82197, 3.33479]	[4.46603, 4.66964]	[0.76757, 0.76997]
NKuIW	[3.96826, 4.49734]	[3.25908, 5.86956]	[4.03131, 4.50760]	[0.71588, 0.82231]
NEGIW	[2.62262, 2.91962]	[4.51696, 4.89758]	[4.69441, 4.90350]	[0.68143, 0.68952]
NLGamIW	[4.79923, 5.41314]	[3.51024, 4.72499]	[4.12389, 4.73166]	[0.79649, 0.92844]
NGoIW	[0.01947, 0.03016]	[0.97378, 1.07021]	[2.40513, 2.43095]	[1.34557, 1.43528]
NIW	[25.97501, 38.82790]	[1.33639, 1.34470]	-----	-----

Table 6 shows the results of the distribution evaluation criteria using -Log, AIC, CAIC, BIC, and HQIC. We note that the NNOWIW distribution has the lowest values in most of these criteria, indicating its superiority and better fit compared to other distributions.

Table 7 shows the results of the statistical goodness-of-fit measures: the Cramer-von-Mises (W) statistic, the Anderson-Darling (A) statistic, and the Kolmogorov-Smirnov (K-S) statistic, in addition to the probability value (p-value) for each distribution.Comparing the values shows that the NNOWIW distribution has the smallest values for W, A, and K-S, with a high p-value (exceeding 0.7), indicating that it is the most suitable model for representing the data. In contrast, other distributions, such as NBeIW and NEGIW, showed higher values for these measures, indicating a poorer fit. The NLGamIW distribution, on the other hand, showed very high values, reflecting its poor fit to the data.

Table 8 displays the estimated intervals for the various model parameters using the maximum likelihood (MLE) method. These parameters include $\eta_{N},\zeta_{N},k_{N},n_{N}$ . It is clear that the NNOWIW distribution has balanced parameter ranges compared to other distributions, reflecting its flexibility and ability to accurately represent data. These results demonstrate that NNOWIW provides a better balance between accuracy and reliability in parameter estimation than competing models.

To further evaluate model adequacy, several goodness-of-fit tests were employed, including the Kolmogorov-Smirnov (K-S), Anderson-Darling (A), and Cramer-von Mises (W) tests. These tests were selected because they capture different aspects of discrepancy between the theoretical and empirical distributions: the K-S test is sensitive to overall deviations, while A and W give more weight to tail behavior. Using multiple goodness-of-fit tests provides a comprehensive and reliable assessment of model performance.

The comparative distributions (NBeIW, NKuIW, NEGIW, NLGanIW, NGoIW, and NIW) were chosen as they represent widely used extensions of the Inverse Weibull family in reliability and lifetime data analysis. Benchmarking the proposed NNOWIW distribution against these flexible alternatives highlights its superior performance in terms of parameter stability and goodness-of-fit, thereby demonstrating its practical applicability to real datasets.

**Figure 4.**
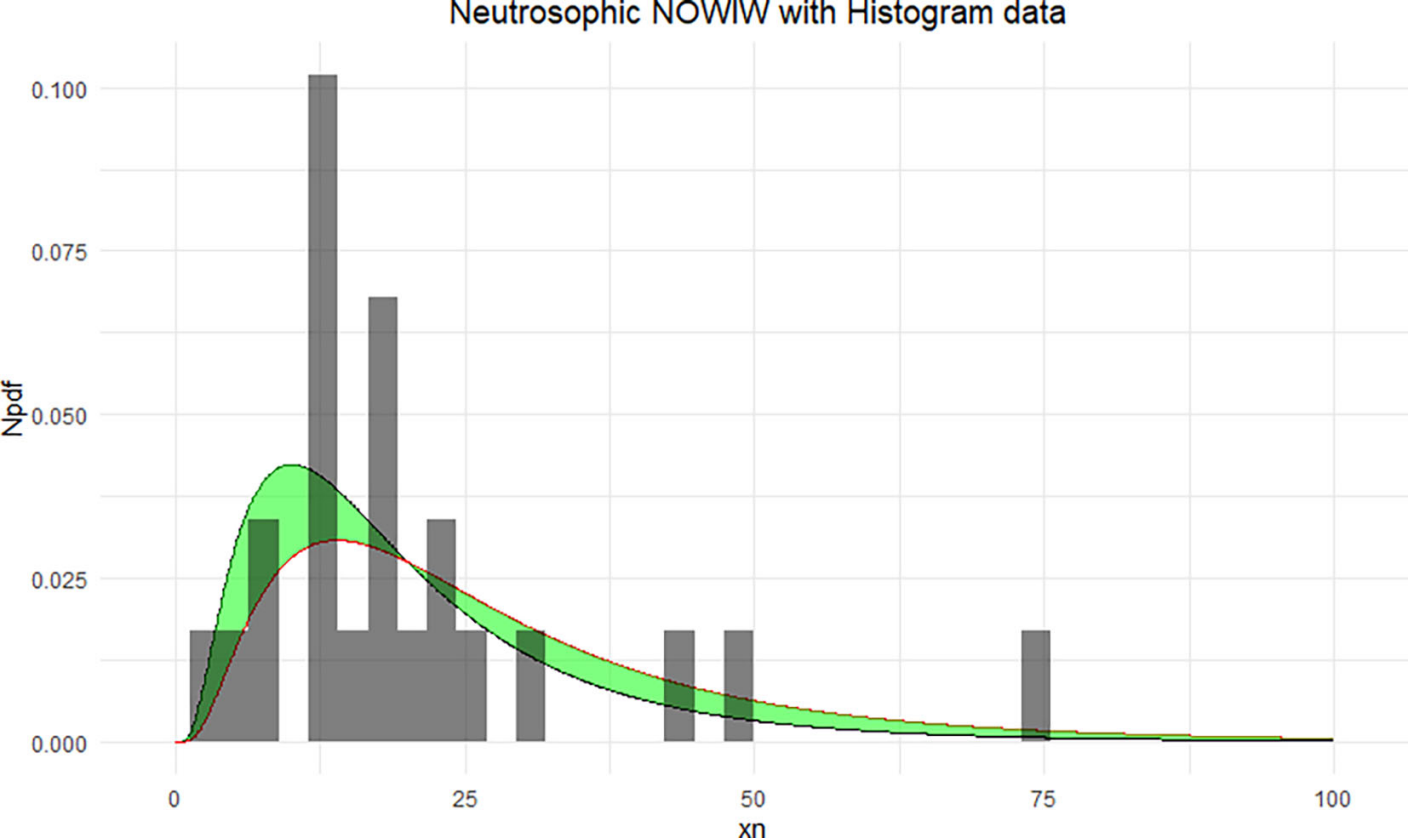
Neutrosophic NOWIW distribution fitted to histogram data.

**Figure 5.**
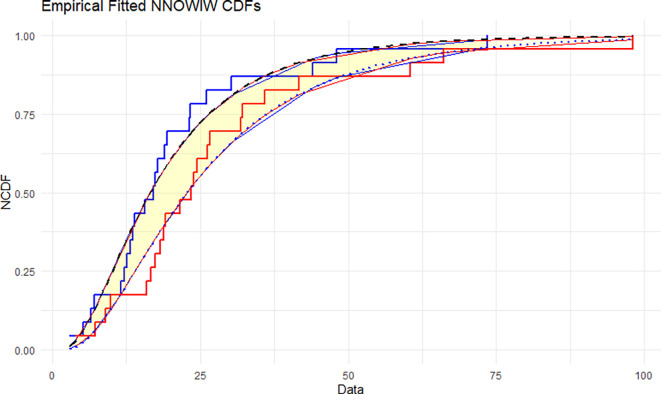
Empirical and fitted NCDFs of the NNOWIW distribution.

The NNOWIW distribution is compared with the experimental histogram data in Figure 4. The fitted neutrosophic model for NNOWIW demonstrates a close fit to the data, effectively capturing variability and uncertainty. The green shaded area indicates the neutrosophic uncertainty range, while the curves (Distribution 1 and Distribution 2) demonstrate the model’s flexibility in adapting to different parameter sets, in Figure 5: The empirical CDFs are compared with the fitted neutrosophic cumulative functions (NCDFs) for the NNOWIW distribution in Figure 5. Both fitted curves follow the steps of the empirical functions exactly with minimal deviation, indicating that the NNOWIW neutrosophic distribution provides an accurate fit to the data and effectively represents its cumulative behavior.

## 7. Conclusion

The study found that the proposed NNOWIW distribution is an effective and flexible model for representing ambiguous and complex data, competitive and accurate performance compared to several competing distributions according to statistical fit criteria and goodness of fit tests. Estimation methods, particularly the maximum likelihood equation MLE, demonstrated their ability to provide accurate parameter estimates with significant improvement as sample size increased. Monte Carlo simulation tests also showed a reduction in bias, MSE, and RMSE, confirming the potential effectiveness of the distribution in statistical analysis of real-world data. A notable strength of the model is its ability to provide interval-based parameter estimates rather than single-point values, which offers a more realistic representation of uncertain or imprecise data. However, the study is limited to data with a medium sample size (23 observations) and unidimensional data, and distributions performance was not tested for multidimensional or multiple failure data.

The scope of the research could be expanded in the future by applying the distribution to larger and more diverse datasets in the engineering, environmental, and medical fields. Further research could involve developing Bayesian versions and robust estimators to improve estimation accuracy in the presence of outliers. In addition, it is recommended to integrate the distribution with hazard models, extend it to the analysis of multiple or sequential failure data, and develop R software packages to facilitate its use in practical applications.

## Data Availability

The study uses a previously published article: *The lifetime in 100 hours of 23 batteries.* https://doi.org/10.1145/3711896.3737372 ^45^ or GitHub - microsoft/BatteryML. ^46^ Interested readers can directly access the dataset. The lifetime in 100 hours of 23 batteries from the cited article using the link above.
