# Supplementary material for: Neutrosophic Extension of the New Odd Weibull-Inverse Weibull Distribution: Theory and Applications
Source: F1000Res. 2026 Apr 17;14:1454. Originally published 2025 Dec 26. [Version 4] doi: 10.12688/f1000research.172480.4 (PMC13169173; doi:10.12688/f1000research.172480.4)
Supplement: Peer review history

# Reviewer report: Reviewer response for version 4

**DOI:** 10.5256/f1000research.198388.r475937

**Published:** 2026-05-07

**Authors:** Mohammad Izat Emir Zulkifly

**Affiliations:** 1 Universiti Teknologi Malaysia (UTM), Johor, Malaysia

The revisions made are appropriate.

Is the work clearly and accurately presented and does it cite the current literature?

Partly

If applicable, is the statistical analysis and its interpretation appropriate?

Partly

Are all the source data underlying the results available to ensure full reproducibility?

Partly

Is the study design appropriate and is the work technically sound?

Partly

Are the conclusions drawn adequately supported by the results?

Partly

Are sufficient details of methods and analysis provided to allow replication by others?

Partly

Reviewer Expertise:

Fuzzy Mathematics, Fuzzy Logic, Geometric Modeling, Computer-Aided Geometric Design (CAGD)

I confirm that I have read this submission and believe that I have an appropriate level of expertise to confirm that it is of an acceptable scientific standard.

# Reviewer report: Reviewer response for version 4

**DOI:** 10.5256/f1000research.198388.r475936

**Published:** 2026-04-27

**Authors:** Bushra Masood

**Affiliations:** 1 University of California, Santa Barbara, California, USA

Although the manuscript has improved, the following problems still require attention:

 1. Figures 1, 2, and 3: Since "Line 1" and "Line 2" do not specify which curve matches which parameter set, the legends are still ambiguous. To make the curves easily distinguishable, the authors should add the actual line styles (such as dashed or solid) next to each parameter specified in the legend.

 2. In the conclusion, since the model has already been created, the first line's usage of the future tense ("will be introduced") is improper. For correctness, this should be changed to the present tense (such as "is introduced").

Is the work clearly and accurately presented and does it cite the current literature?

Partly

If applicable, is the statistical analysis and its interpretation appropriate?

Partly

Are all the source data underlying the results available to ensure full reproducibility?

Yes

Is the study design appropriate and is the work technically sound?

Yes

Are the conclusions drawn adequately supported by the results?

Partly

Are sufficient details of methods and analysis provided to allow replication by others?

Yes

Reviewer Expertise:

Statistical modeling, probability distributions, distribution theory, neutrosophic statistics, directional statistics, simulation studies, parameter estimation, goodness-of-fit analysis, machine learning, and time series analysis.

I confirm that I have read this submission and believe that I have an appropriate level of expertise to confirm that it is of an acceptable scientific standard.

# Reviewer report: Reviewer response for version 3

**DOI:** 10.5256/f1000research.197567.r470290

**Published:** 2026-03-28

**Authors:** Bushra Masood

**Affiliations:** 1 University of California, Santa Barbara, California, USA

To account for uncertainty through an indeterminate component, the paper suggests a neutrosophic extension of the Odd Weibull–Inverse Weibull distribution.

 Together with simulation research and an actual data application, the authors define the characteristics of the suggested model and use graphical representations to show its behavior. Though several parts of the book need to be clarified and improved, the concept of integrating uncertainty within a neutrosophic framework is intriguing and pertinent.

 Although the definition of the neutrosophic component is ambiguous and varied, the presentation is typically intelligible. Specifically, there is confusion since the indeterminate term t _I_ is presented using distinct notations (e.g., t _I_ ∈ [XL, XU] and subsequently �� _I_ ∈ [����, ����]). This component should be defined clearly and consistently by the writers, who should also use consistent notation throughout.

 Furthermore, there is room for development in the graphical depiction. The usage of "Line 1" and "Line 2" in Figures 1, 2, and 3 is conceptually inappropriate because the model is interval-based. Instead, the authors should provide parameter intervals and use appropriate labeling to fully explain them in the figure legend.

 Additionally, comparisons with current distributions should be included in the application section's fitted PDF and empirical CDF displays. This would make it easier to evaluate the model's performance and show if it is better than conventional options.

 Lastly, the conclusion essentially restates the findings and should be improved by emphasizing the key contributions, going over the practical ramifications, and offering potential avenues for future study.

 Overall, the paper offers a potential concept; nevertheless, to increase the work's scientific quality, the problems with the description of the neutrosophic component, figure clarity, lack of comparative analysis in the application, and a weak conclusion should be fixed.

Is the work clearly and accurately presented and does it cite the current literature?

Partly

If applicable, is the statistical analysis and its interpretation appropriate?

Partly

Are all the source data underlying the results available to ensure full reproducibility?

Yes

Is the study design appropriate and is the work technically sound?

Yes

Are the conclusions drawn adequately supported by the results?

Partly

Are sufficient details of methods and analysis provided to allow replication by others?

Yes

Reviewer Expertise:

Statistical modeling, probability distributions, distribution theory, neutrosophic statistics, directional statistics, simulation studies, parameter estimation, goodness-of-fit analysis, machine learning, and time series analysis.

I confirm that I have read this submission and believe that I have an appropriate level of expertise to confirm that it is of an acceptable scientific standard, however I have significant reservations, as outlined above.

# Reviewer report: Reviewer response for version 3

**DOI:** 10.5256/f1000research.197567.r464962

**Published:** 2026-03-26

**Authors:** Mohammad Izat Emir Zulkifly

**Affiliations:** 1 Universiti Teknologi Malaysia (UTM), Johor, Malaysia

The paper proposes a neutrosophic extension of the new odd Weibull inverse Weibull family, derives its distributional functions and several theoretical properties, studies parameter estimation via MLE/LSE/WLSE, evaluates estimator performance by Monte Carlo simulation, and illustrates the model with one battery lifetime dataset of 23 interval-valued observations. The paper itself also states that broader validation on larger and more diverse datasets is still needed. The manuscript addresses a familiar genre of statistical distribution paper: it introduces a new model, derives its main functions, studies analytical properties, proposes estimation procedures, runs a simulation study, and compares the model with competing distributions on real data. The stated motivation is that many generated lifetime families assume exact observations, whereas neutrosophic logic is intended to represent ambiguity, indeterminacy, and interval-valued uncertainty more explicitly. The article organizes this work into sections on formulation, properties, estimation, simulation, application, and conclusion. Version 3 also notes that the authors revised the empirical section to clarify that there is only one real-data application, clarified the rationale for parameter values, and revised the simulation to better align with the size of the real dataset.

 In principle, that is a reasonable and indexable structure for a methodological paper in applied probability or mathematical statistics. However, in its present form, the manuscript is still only **partly** satisfactory on all six questions. The main issues are not that the paper lacks an idea, but that it lacks enough clarity, rigor, and reproducibility to make the contribution fully convincing. Many equations and symbols are hard to follow, the notation is inconsistent, the estimation and simulation procedures are not described in enough operational detail for straightforward replication, and the empirical support is thin because it relies on a single small battery dataset. Those limitations do not necessarily invalidate the paper, but they do mean the paper still requires substantial revision before it can be considered scientifically robust.

 My overall recommendation would be **major revision**. The topic is potentially suitable for indexing, but several scientific and presentation issues must be resolved before the article can be considered sound and reproducible.

 1. Is the work clearly and accurately presented and does it cite the current literature?

**Answer: Partly**

 The manuscript is presented in a recognizable scholarly structure, and it does cite both foundational and recent references. The introduction situates the work within failure-time modeling, T-X generated families, and neutrosophic logic, and the abstract and section outline are coherent at a high level. The paper also includes recent material up to 2025–2026, so the literature is not obviously outdated.

 That said, the presentation is only partly clear. The main weakness is the manuscript’s readability and notation. Across the formulation and properties sections, many equations are difficult to parse, symbols are not always introduced cleanly, and the notation shifts between expressions in a way that makes it hard to verify derivations line by line. There are also noticeable language problems, typographical issues, and local inconsistencies, such as the simulation heading referring to “NMWIW” rather than NNOWIW. These are not merely cosmetic problems: in a theoretical statistics paper, notation and exposition are part of the scientific content because readers need them to check correctness. The authors should perform a full technical rewrite focused on precision and consistency. In particular:

 - Add a notation table defining every symbol once and using it consistently throughout.

 - Re-typeset and re-check every equation, especially in Sections 2-4, so the notation is readable and internally consistent.

 - Ensure every derived quantity is explicitly tied to the preceding definition.

 - Revise the English carefully, ideally with help from a fluent scientific editor.

 - Remove typographical inconsistencies and section-title errors.

 - Strengthen the literature review by distinguishing more clearly what is genuinely new here relative to other neutrosophic extensions and inverse-Weibull-based generalizations.

 - Clear, correct notation and readable derivations are required, not optional. If the mathematical objects cannot be followed unambiguously, the paper cannot be reliably assessed.

 2. Is the study design appropriate and is the work technically sound?

**Answer: Partly**

 The high-level design is appropriate for this kind of paper. The authors define a new family, derive NCDF/NPDF/survival/hazard functions, study properties such as quantiles, moments, entropy, and related functionals, then move to estimation, simulation, and one application. That overall design is standard and acceptable for a methodological distribution paper.

 However, technical soundness is only partly established. The theoretical development is extensive, but the current presentation does not always make it possible to verify whether all derivations are correct. Several formulas are written too compactly, some depend on expansions whose convergence conditions or validity domains are not fully discussed, and the transition from classical to neutrosophic interval-valued parameters is asserted more often than it is rigorously operationalized. The manuscript also claims practical flexibility based largely on figures, summary tables, simulation, and one application, but that is not enough by itself to establish broad technical superiority.

 A second issue is that the empirical and validation side is thin. The paper now explicitly says there is only one real-data application, and that application uses just 23 batteries. That is acceptable as an illustration, but weak as evidence for strong claims about general usefulness. The abstract itself recognizes that larger and more diverse datasets are still needed. The authors should strengthen technical soundness as follows:

**- **Add formal verification steps for key derivations, especially those used later in estimation and simulation.

 - State assumptions explicitly, including parameter domains, regularity conditions, and any conditions needed for moment existence, expansion validity, and estimator behavior.

 - Explain what the neutrosophic extension changes in practice, beyond replacing scalar parameters with intervals.

 - Broaden the validation, ideally with more than one real dataset and with comparisons to stronger benchmark models.

 - The paper must make the mathematical derivations verifiable and must justify the neutrosophic construction in a technically explicit way. Without that, the paper remains scientifically fragile.

 3. Are sufficient details of methods and analysis provided to allow replication by others?

**Answer: Partly**

 The manuscript provides the general formulas for the proposed model and names the estimation methods used: MLE, LSE, and WLSE. It also reports that a Monte Carlo study was conducted with 1000 iterations at sample sizes 20, 50, 100, and 200, using specified parameter intervals. That gives readers the general outline of the workflow.

 But replication requires much more than a conceptual outline. At present, the paper does not provide enough implementation detail for another researcher to reproduce the results reliably. The missing or under-specified elements include:

 - how random samples were generated from the proposed distribution in the simulation;

 - which optimization algorithm was used for MLE/LSE/WLSE;

 - how interval-valued parameters were handled computationally;

 - what starting values, bounds, stopping criteria, and convergence checks were used;

 - whether failed optimizations occurred and how they were treated;

 - what software and code environment were used;

 - how goodness-of-fit statistics and p-values were computed in the application.

 The authors should add a dedicated Replication and Implementation subsection containing:

 - pseudocode or a stepwise algorithm for simulation and estimation;

 - the exact random-generation method from the quantile function or another sampler;

 - software details, package names, and version numbers;

 - optimization details, including initial values and constraints;

 - how interval outputs were summarized;

 - complete formulas and computational details for all fit criteria and goodness-of-fit tests;

 - a public code repository or supplementary code appendix.

 - Reproducibility details are required. A methodological paper that cannot be independently rerun is not yet scientifically adequate.

 4. If applicable, is the statistical analysis and its interpretation appropriate?

**Answer: Partly**

 This question is applicable. The paper includes estimator comparison by simulation and model fitting to data using information criteria and goodness-of-fit measures. In broad terms, these are reasonable tools for a paper of this type. The authors compare MLE, LSE, and WLSE using RMSE and bias, and they compare the proposed model to competitors using AIC/CAIC/BIC/HQIC and several fit statistics.

 The problem is that the statistical analysis is not yet reported with enough depth or caution. The simulation study uses only one parameter setting and a limited set of sample sizes, and the application is based on just one dataset with 23 interval observations. The manuscript then interprets the findings rather strongly, especially the claim that MLE is the “best” and that the proposed model is strongly competitive. Those may be plausible conclusions within the limited examples shown, but they are not yet general conclusions. Also, the paper gives formulas for p-values and test statistics, but the excerpted application details are incomplete, and the manuscript needs to show the actual test outputs transparently, not just summary claims. The authors should improve the statistical analysis by:

 - expanding the simulation to include multiple parameter scenarios, not just one;

 - reporting Monte Carlo variability more fully, such as standard errors or confidence intervals for performance measures;

 - discussing numerical instability or convergence failures, if any;

 - including the full application results table, with all fit statistics and p-values clearly presented;

 - adding at least one or two more real datasets, preferably from different contexts;

 - tempering interpretation so conclusions match the scale of the evidence.

 - At minimum, the paper must report the statistical procedures and outputs transparently and avoid overgeneralizing from one small application. Those points are required.

 5. Are all the source data underlying the results available to ensure full reproducibility?

**Answer: Partly**

 The manuscript does present the battery lifetime data in the application section, at least in part, and indicates that the real data consist of interval-valued lifetimes for 23 batteries. That is a positive step.

 Still, the answer is only partly. Full reproducibility requires more than partial inclusion of the data in the manuscript text. The reader should have access to:

 - the complete dataset in machine-readable form;

 - the exact formatting used for analysis;

 - the code that produced the parameter estimates, fit criteria, goodness-of-fit results, figures, and simulation tables.

 At present, the manuscript does not provide a complete reproducibility package. Even if the data are visible in the article, the absence of accompanying code and detailed computational files means another researcher may not be able to reproduce Tables 1-3, the application results, or the figures exactly.

 The authors should provide:

 - the full battery dataset as supplementary CSV or text;

 - all simulation scripts;

 - all estimation and plotting scripts;

 - a README describing how to reproduce every table and figure;

 - ideally, a permanent public repository or journal supplement.

 - A complete reproducibility package is strongly recommended, and at minimum the full dataset and enough implementation detail to regenerate the main results are required.

 6. Are the conclusions drawn adequately supported by the results?

**Answer: Partly**

 The conclusions are directionally consistent with the work shown. The paper demonstrates that the proposed distribution is mathematically flexible, can produce varied shapes in its functions and plots, and performs competitively under the reported simulation and the single battery-data application. The abstract also appropriately adds a caution that broader validation is still needed.

 However, the evidence base is still too narrow to support stronger general claims. One simulation setup and one small dataset do not establish that the model is broadly preferable, generally superior, or widely applicable across reliability problems. The strongest defensible conclusion at this stage is more limited: the model appears promising and worthy of further study, not yet definitively established as a superior practical model. The authors should revise the conclusion so that it:

 - distinguishes theoretical contribution from empirical validation;

 - states clearly that current empirical evidence is preliminary;

 - avoids broad superiority claims unless supported by more applications and stronger benchmarking;

 - explains what future work is needed to establish general usefulness.

 - The conclusions must be narrowed to what the results actually show. This is required.

 The following issues should be treated as mandatory revisions:

 1. Mathematical clarity and notation

 The equations, symbols, and derivation flow must be made unambiguous and internally consistent. At present, the formulation and properties sections are too difficult to verify reliably.

 2. Reproducible estimation and simulation details

 The paper must specify exactly how data were simulated, how parameters were estimated, what algorithms and software were used, and how interval-valued parameters were computed in practice.

 3. Transparent empirical reporting

 The application section must show the full dataset, the full comparative results, and the full goodness-of-fit outputs, including p-values and model-estimation details.

 4. Appropriate interpretation of evidence

 The claims must be aligned with the actual validation scope. With only one small real dataset, the conclusions should be presented as preliminary.

 5. Language and technical editing

 The manuscript requires thorough editing for grammar, notation consistency, and typographical errors because these currently interfere with scientific interpretation.

 Final overall assessment

 This manuscript has a potentially indexable core idea and follows a generally appropriate structure for a distribution theory paper. However, it is not yet strong enough in clarity, verification, reproducibility, and empirical support to merit an unqualified positive assessment. My answers remain **Partly** for all six questions. With substantial revision especially improved derivational clarity, explicit computational detail, complete reproducibility materials, and more careful interpretation, the article could become scientifically sound. At present, it still requires major revision.

Is the work clearly and accurately presented and does it cite the current literature?

Partly

If applicable, is the statistical analysis and its interpretation appropriate?

Partly

Are all the source data underlying the results available to ensure full reproducibility?

Partly

Is the study design appropriate and is the work technically sound?

Partly

Are the conclusions drawn adequately supported by the results?

Partly

Are sufficient details of methods and analysis provided to allow replication by others?

Partly

Reviewer Expertise:

Fuzzy Mathematics, Fuzzy Logic, Geometric Modeling, Computer-Aided Geometric Design (CAGD)

I confirm that I have read this submission and believe that I have an appropriate level of expertise to state that I do not consider it to be of an acceptable scientific standard, for reasons outlined above.

# Reviewer report: Reviewer response for version 2

**DOI:** 10.5256/f1000research.196473.r462130

**Published:** 2026-03-05

**Authors:** Salah Abid

**Affiliations:** 1 Mustansiriyah University, Baghdad, Baghdad Governorate, Iraq

Dear editor in chief,

 There are still fundamental errors in the manuscript that cannot be ignored:

 1. The selection of the default parameters was not based on scientific criteria but was arbitrary. It would be better to choose parameters that reflect different patterns of the distribution so that the recommendations represent a wide range of phenomena.

 2. The manuscript mentions two applications based on real data, while in reality, we only find one application based on real data.

 3. The sample sizes chosen for the simulation are more than or equal to 50, while the sample size used in the application is 23. This creates a disconnect between the applied and experimental (simulation) aspects, thus preventing the application from utilizing the simulation results.

 Professor Salah H Abid (PhD)

Is the work clearly and accurately presented and does it cite the current literature?

Yes

If applicable, is the statistical analysis and its interpretation appropriate?

Not applicable

Are all the source data underlying the results available to ensure full reproducibility?

Partly

Is the study design appropriate and is the work technically sound?

Partly

Are the conclusions drawn adequately supported by the results?

Partly

Are sufficient details of methods and analysis provided to allow replication by others?

Partly

Reviewer Expertise:

statistical distributions, data analysis, models building

I confirm that I have read this submission and believe that I have an appropriate level of expertise to confirm that it is of an acceptable scientific standard, however I have significant reservations, as outlined above.

# Response

**Published:** 2026-03-09

**Authors:** Nooraldeen Alsaab

**Affiliations:** Mathematics, Anbar General Directorate of Education, Ministry of Education, Fallujah, Iraq

**Response to the Reviewer**

 We sincerely thank the Reviewer for the careful reading of the manuscript and for pointing out these important issues. We have revised the manuscript accordingly.

**Comment 1:** *The selection of the default parameters was not based on scientific criteria but was arbitrary. It would be better to choose parameters that reflect different patterns of the distribution so that the recommendations represent a wide range of phenomena.*

**Response:**

 Thank you for this important observation. We revised the discussion accompanying Figures 1–3 to clarify the rationale for selecting the neutrosophic parameter intervals. The parameters were not intended as arbitrary values; rather, they were chosen to represent different levels of uncertainty and to generate distinct distributional patterns, including more concentrated shapes, wider spread, and heavier-tail behavior. This clarification has now been added explicitly in the revised manuscript to better justify the selected intervals and to show that they were used to illustrate the flexibility of the proposed model across multiple distributional behaviors.

**Comment 2:** *The manuscript mentions two applications based on real data, while in reality, we only find one application based on real data.*

**Response:**

 We appreciate the Editor for identifying this inconsistency. The manuscript has been corrected to state clearly that the paper contains one real-data application only, based on the battery lifetime dataset. The previous wording referring to “two real-world datasets” was inaccurate and has been revised throughout the manuscript to avoid any ambiguity and to ensure full consistency between the text and the actual empirical analysis presented.

**Comment 3:** *The sample sizes chosen for the simulation are more than or equal to 50, while the sample size used in the application is 23. This creates a disconnect between the applied and experimental aspects.*

**Response:**

 Thank you for this valuable comment. In response, we revised the simulation study to reduce the gap between the Monte Carlo design and the empirical application. The simulation settings were updated to include small-sample scenarios closer to the real-data application size, so that the behavior of the estimators can be assessed under conditions more comparable to the empirical dataset. We also revised the discussion of the simulation section to emphasize this connection more clearly. This modification improves the coherence between the experimental and applied parts of the manuscript and makes the simulation findings more relevant to the real-data analysis.

# Reviewer report: Reviewer response for version 1

**DOI:** 10.5256/f1000research.190211.r446473

**Published:** 2026-02-10

**Authors:** Salah Abid

**Affiliations:** 1 Mustansiriyah University, Baghdad, Baghdad Governorate, Iraq

The research is well-written. I suggest reconsidering the estimation methods and using only the maximum likelihood method to describe the behavior of model parameters, as the other methods used here are outdated and unnecessary to mention; there are better and more modern methods available.

 The p-values of the Cramer-von-Mises (W) and the Anderson-Darling (A) tests are not found in table (7). Those values ​​should be placed in the table.

 1. The data that is supposed to be factual, as indicated in the manuscript, is not found in the manuscript itself, nor in the source cited in the manuscript as containing that data. Therefore, I have serious doubts about the practical application and the results obtained in the manuscript, as the data in this case is fictitious and untrue. If this is the case, then the results will be fabricated. Please include the data in the manuscript so that we can review it.

 2. The manuscript requires significant revisions before it can be considered to be fully scientifically valid. (Approved with Reservations)

Is the work clearly and accurately presented and does it cite the current literature?

Yes

If applicable, is the statistical analysis and its interpretation appropriate?

Not applicable

Are all the source data underlying the results available to ensure full reproducibility?

Partly

Is the study design appropriate and is the work technically sound?

Partly

Are the conclusions drawn adequately supported by the results?

Partly

Are sufficient details of methods and analysis provided to allow replication by others?

Partly

Reviewer Expertise:

statistical distributions, data analysis, models building

I confirm that I have read this submission and believe that I have an appropriate level of expertise to confirm that it is of an acceptable scientific standard, however I have significant reservations, as outlined above.
